# Proteolytic Processing, Maturation, and Unique Synteny of the *Streptomyces* Hemagglutinin SHA

**DOI:** 10.1128/Spectrum.00766-21

**Published:** 2021-09-01

**Authors:** Yoko Fujita-Yamaguchi, Hideyuki Muramatsu, Alonso Tapia, Karine Bagramyan, Moksha Desai, Yasuhiro Takehana, Masayuki Igarashi, Yoshiki Yamaguchi, Markus Kalkum

**Affiliations:** a Department of Diabetes Complications and Metabolism, Arthur Riggs Diabetes and Metabolism Research Institute, Beckman Research Institute, City of Hope, Duarte, California, USA; b Department of Immunology and Theranostics, Arthur Riggs Diabetes and Metabolism Research Institute, Beckman Research Institute, City of Hope, Duarte, California, USA; c Laboratory of Microbiology, Institute of Microbial Chemistry, Tokyo, Japan; d Biomolecular Characterization Unit, RIKEN Center for Sustainable Resource Science, Saitama, Japan; Emory University School of Medicine

**Keywords:** microbial lectin, *Streptomyces*, blood type B-specific hemagglutination, lectin synteny

## Abstract

SHA is an l-rhamnose- and d-galactose-binding lectin that agglutinates human group B erythrocytes and was first purified almost 50 years ago. Although the original SHA-producing *Streptomyces* strain was lost, the primary structure of SHA was more recently solved by mass spectrometry of the archived protein, which matched it to a similar sequence in the Streptomyces lavendulae genome. Using genomic and protein biochemical analyses, this study aimed to identify SHA-secreting *Streptomyces* strains to further investigate the expression and binding activities of these putative proteins. Of 67 strains genetically related to *S. lavendulae*, 17 secreted pro-SHAs in culture. Seven SHA homologues were purified to homogeneity and then subjected to liquid chromatography–high-resolution multistage mass spectrometry (LC-MS/MS) and hemagglutination (HA) assays. Processing of pro-SHAs occurred during and after purification, indicating that associated proteases converted pro-SHAs into mature SHAs with molecular masses and HA activities similar to that of the archived SHA. Previously, the SHA monomer was shown to have two carbohydrate binding sites. The present study, however, found no HA activity in pro-SHAs, suggesting that pro-SHAs have only one binding site. Genetically, the SHA gene resides in conserved syntenic regions. The published genomes of 1,234 *Streptomyces* strains were analyzed, revealing 18 strains with SHA genes, 16 of which localized to a unique syntenic region. The SHA syntenic region consists of ∼17 open reading frames (ORFs) and is specific to *S. lavendulae*-related strains. Notably, a lipoprotein gene excludes SHA from the synteny in some strains, suggesting that horizontal gene transfer events during the course of evolution shaped the distribution of SHA genes.

**IMPORTANCE** Lectins are extremely useful molecules for the study of glycans and carbohydrates. Here, we show that homologous genes encoding the l-rhamnose- and d-galactose-binding lectins, SHAs, are present in multiple bacterial strains, genetically related to Streptomyces lavendulae. SHA genes are expressed as precursor pro-SHA proteins that are truncated and mature into fully active lectins with two carbohydrate binding sites, which exhibit hemagglutination activity for type B red blood cells. The SHA gene is located within a conserved syntenic region, hinting at specific but yet-to-be-discovered biological roles of this carbohydrate-binding protein for its soil-dwelling microbial producer.

## INTRODUCTION

The blood type B-specific hemagglutinin (HA) secreted by *Streptomyces* sp. strain 27S5 (SHA) was purified and characterized over 40 years ago (1–4). The original *Streptomyces* sp. strain 27S5 was lost long ago, and therefore no genomic information is available; however, the amino acid sequence of its freezer-preserved SHA was recently determined by mass spectrometry, matching a hypothetical protein in the genome of Streptomyces lavendulae ATCC 14158 (*Lav*) (5). The SHA homologue of *S. lavendulae* ATCC 14158 had over 99% homology to the authentic archived SHA and contained one amino acid difference: residue 108 was a glutamic acid in authentic archived SHA and an alanine in its *S. lavendulae* homologue (5). Recombinant SHA (rSHA) consisting of the SHA homologous domain with a replacement of alanine by glutamic acid was expressed and purified. The binding of rSHA to l-rhamnose on microbial cells and blood type B specificity were confirmed by staining of Lactobacillus casei (Shirota) and by glycan microarray analyses, respectively, supporting the notion that rSHA is equivalent to the authentic archived SHA (5). A notable difference in the hypothetical protein of *S. lavendulae* ATCC 14158 was that 68 amino acids preceded the homologous SHA domain at its N terminus. In fact, of 11 SHA homologous (hypothetical) proteins found in databases in 2016, all contained such N-terminal domains (5). However, such additional amino acids at the N terminus were not detected in the authentic archived SHA. This indicated that SHA was most likely matured from pre- and pro-protein forms. Another puzzling initial observation was that when *S. lavendulae* and *Streptomyces* sp. Mg1 strains, whose genomes encode hypothetical SHA proteins, were cultured under the conditions previously used (2, 3), none of them appeared to express SHA homologues, based on the lack of HA activity of their culture broths (5).

To address questions about how SHA was biosynthesized and secreted by the lost strain *Streptomyces* sp. 27S5 as well as what biological roles SHA may have, we needed to identify SHA-secreting *Streptomyces* strains that are identical to or at least comparable to the lost strain. To start this project, we took advantage of the collection of over 40,000 actinomycete strains of the Institute of Microbial Chemistry (IMC). Of 5,000 strains with available 16S rRNA gene signatures, 67 strains with significant 16S rRNA sequence homology to that of *S. lavendulae* were examined. Based on positive results of Western blotting and PCR analyses, six strains were chosen for purification and characterization. The SHA domains of these homologues and those of an additional four strains were subjected to PCR amplification sequencing of putative SHA genes, which revealed amino acid sequences of 10 SHA homologues. Here, we show liquid chromatography–high-resolution multistage mass spectrometry (LC-MS/MS) analyses of six purified SHA homologues and match them to the corresponding deduced amino acid sequences derived from the determined DNA sequences. These experiments revealed the amino acid sequences of pro-SHA proteins and processing sites for yet-to-be identified proteases, which must be responsible for producing mature SHA proteins. Furthermore, the expression of hypothetical SHA homologues was detected in culture supernatants from *S. lavendulae* and *Streptomyces* sp. Mg1 by Western blotting using anti-SHA rabbit serum. The SHA homologue was purified from the culture supernatant of *S*. *lavendulae* ATCC 14158 (abbreviated *Lav*) by gum arabic affinity chromatography. The *Streptomyces* sp. Mg1 SHA homologue, however, did not bind to gum arabic gels and was therefore not purified. All seven purified SHA homologues were compared to the authentic archived SHA in terms of expression/secretion levels and HA activities.

Although the current approach taken was to find strains carrying SHA homologues by narrowing down target strains based on the 16S rRNA signature of *S. lavendulae*, which resulted in successful purification and characterization of SHA proteins, a low correlation between 16S rRNA and SHA homologues by phylogenetic analyses became obvious in this study. Thus, a new genomic screening for SHA homologues was carried out. Comparative analyses of 18 SHA homologue genes found in 1,234 *Streptomyces* strains downloaded from the NCBI database revealed that 16 SHA homologue genes are in novel syntenic regions which are distributed specifically among *S. lavendulae*-related strains.

## RESULTS

### Narrowing down of candidate *Streptomyces* strains that likely produce SHA homologues.

**FIG 1:**
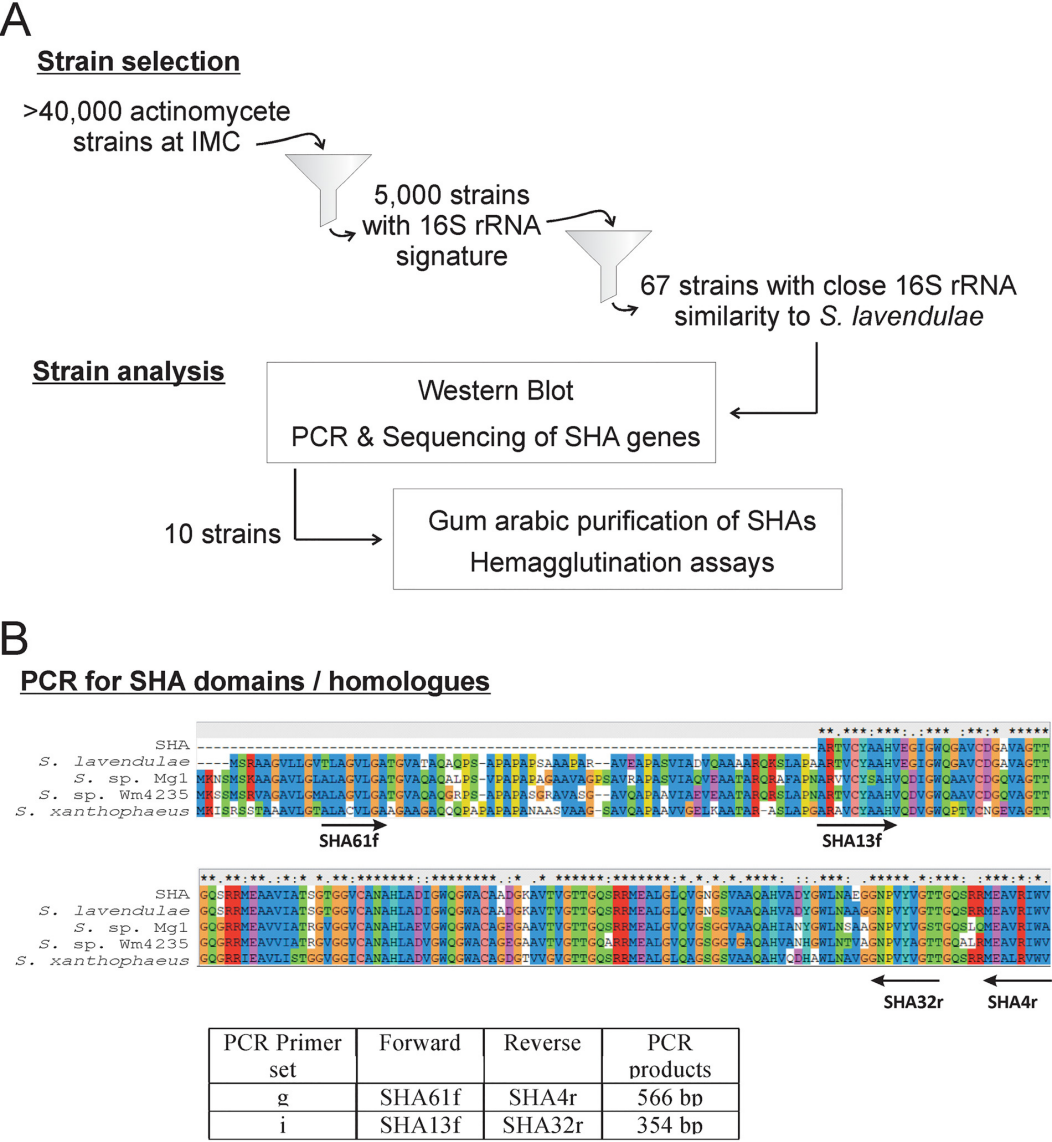
(A) Flow chart of the experiments performed in this study. (B) Sequences of SHA homologues marking the relative locations of primers used to amplify SHA genes. The primer sequences listed in Table 1 were designed based on the *S. lavendulae* DNA sequence (7). Shown are the locations of PCR primers relative to amino acid sequences of the authentic SHA, the *S. lavendulae* SHA homologue, and three other SHA homologues (*Streptomyces* sp. Mg1, *Streptomyces* sp. Wm4235, and *S. xanthophaeus*), as previously reported (5).

Our strategy is outlined in Fig. 1A. IMC stocks over 40,000 actinomycete strains. Of 5,000 strains with available 16S rRNA gene signatures, we selected 67 strains that had 99.6% to 100% 16S rRNA sequence homology to that of *S. lavendulae*. Based on the DNA sequence of the *S. lavendulae* SHA homologue, PCR primers were designed. Various sets of forward and reverse primers were used to determine whether they yield predicted PCR products. PCR amplifications of SHA homologues were carried out using the best primer sets, g and i, which produced PCR products of 566 and 354 bp, respectively (Fig. 1B; Table 1). Of 67 strains tested, 11 yielded PCR products for both primer sets g and i, while 24 were positive for primer set g but gave only faint PCR product bands for primer set i. The results of PCR experiments along with information on 67 IMC strains are listed in Table S1 in the supplemental material.

**TABLE 1:** Primers used in this study

Target	Primer name	Positions (nt)	DNA sequence	Detection	Sequencing	Note
16S rRNA	16S001F	6–27	5′-GGAGAGTTTGATCCTGGCTCAG-3′	√		
	16S003R	1512–1491	5′-ACGGCTACCTTGTTACGACTTC-3′	√		
	9f	9–27	5′-GAGTTTGATCCTGGCTCAG-3′		√	
	338F	338–357	5′-ACTCCTACGGGAGGCAGCAG-3′		√	
	536/517R	536–517	5′-GTATTACCGCGGCTGCTGGC-3′		√	
	907/928F	907–928	5′-AAACTCAAAGGAATTGACGGGG-3′		√	
SHA homologue	SHA61f	47–68	5'-CGCTGGCGGGGGTGCTSGGNGC-3'	√	√	Set g
	SHA4r	612–588	5'-TCAGACCCAGATGCGGACGGCYTCC-3'	√	√	Set g
	SHA13f	217–244	5'-CCCGGACGGTCTGTTACGCCGCNCAYG-3'	√	√	Set i
	SHA32r	570–544	5'-GTGGTGGCCCACGTAGACCGGRTTNCC-3'	√	√	Set i
	SHA63f	40–68	5'-GGATGGCGCTGGCGGGGGTGCTSGGNGC-3'	√	√	For strain #26
	SHA5f	383–407	5'-TCGGCTGGCAGGGCTGGGCNTGYGC-3'		√	
	SHA24r	446–421	5'-TGGCCCGTGGTGCCGACCGTSACNGC-3'		√	
	SHA24r26	446–421	5′-TGCCCGGTGGTGCCCACCGTCACGGC-3′		√	For strain #26

**FIG 2:**
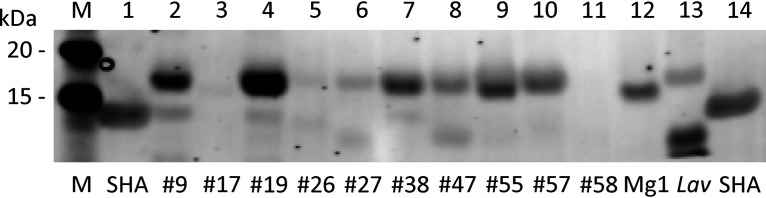
Western blot analysis of SHA homologue proteins in culture supernatants of 12 *Streptomyces* strains detected by rabbit anti-SHA serum. Ten IMC strains listed in Table 2 (#9, #17, #26, #39, #47, #55, #57, and #58) as well as *Streptomyces* sp. strain Mg1 and *S. lavendulae* (*Lav*; ATCC 14158), which were previously studied (5), were cultured under the same conditions as described in Materials and Methods. SHA purified over 40 years ago (3) was included as a positive control (0.1 μg in lanes 1 and 14). Precision Plus Protein Kaleidoscope standards (Bio-Rad 161–0375) were used as markers (lane M). A portion of the immunoblot showing the 20- and 15-kDa area is shown.

Western blotting (WB) of culture supernatants, which had been concentrated 4-fold by trichloroacetic acid (TCA) precipitation, revealed that 17 IMC *Streptomyces* strains that were PCR positive with primer sets g and i produced SHA homologues with molecular masses greater than that of the authentic archived SHA. A typical WB result for 10 IMC strains is shown in Fig. 2. It is notable, however, that culture supernatants of strains #9, #19, and #38 contained minor bands, with molecular masses similar to that of the authentic archived SHA. Of strains #17, #26, and #58, which gave PCR products with primer set g but were inconclusive with primer set i, #58 did not lead to any immunodetectable bands (Fig. 2; Table 2). Although strains #17 and #26 showed very faint bands in Fig. 2, the expression of SHA homologues was confirmed by independent WB analyses (data not shown). To ensure that purification of SHA homologues by gum arabic affinity chromatography can be achieved, the supernatants were incubated with gum arabic gels, and the captured SHA analogues were dissolved in SDS-PAGE sample buffer for WB analysis. Using the positive results shown in Table 2, six *Streptomyces* strains were chosen for purification.

**TABLE 2:** Summary of *Streptomyces* strains used in this study

IMC strain used^*a*^	Strain description^*b*^	Result^*c*^ for:		
		PCR using primer sets g and i	WB using TCA-precipitated culture broths	WB using gum arabic precipitates
#9	*Streptomyces* sp. 3015-22	+ and +	+	+
#17	*Streptomyces* sp. 3020-26	+ and ±	±	±
#19	*Streptomyces* sp. 3024-17	+ and +	+	+
#26	*Streptomyces* sp. isolate 491	+ and ±	+	+
#27	*S. lavendulae* ISP 5069^T^	+ and +	+	+
#38	*Streptomyces* sp. BSA00614	+ and +	+	+
#47	*Streptomyces* sp. BSA00904	+ and +	+	ND
#55	*Streptomyces* sp. BSA01223	+ and +	+	ND
#57	*Streptomyces* sp. BSA01241	+ and +	+	+
#58	*Streptomyces* sp. BSA01241	+ and ±	−	ND

a Designations of IMC strains #1 to #67 studied and abbreviation used.

b Further details of the strains, including accession numbers for 16S RNA and SHA homologues, are listed in Table 7.

c The intensities of bands are semiquantitatively described as distinctive (+) and faint (±). ND, not determined.

All the results obtained by PCR and WB for the *Streptomyces* strains examined in this study are summarized in Table 2. Candidate strains for purification of SHA homologues were thus narrowed down to six (strains #9, #19, #26, #27, #38, and #57) from the 67 IMC strains (Table S1).

### Purification and determination of the amino acid sequences of SHA homologues from six *Streptomyces* strains.

**FIG 3:**
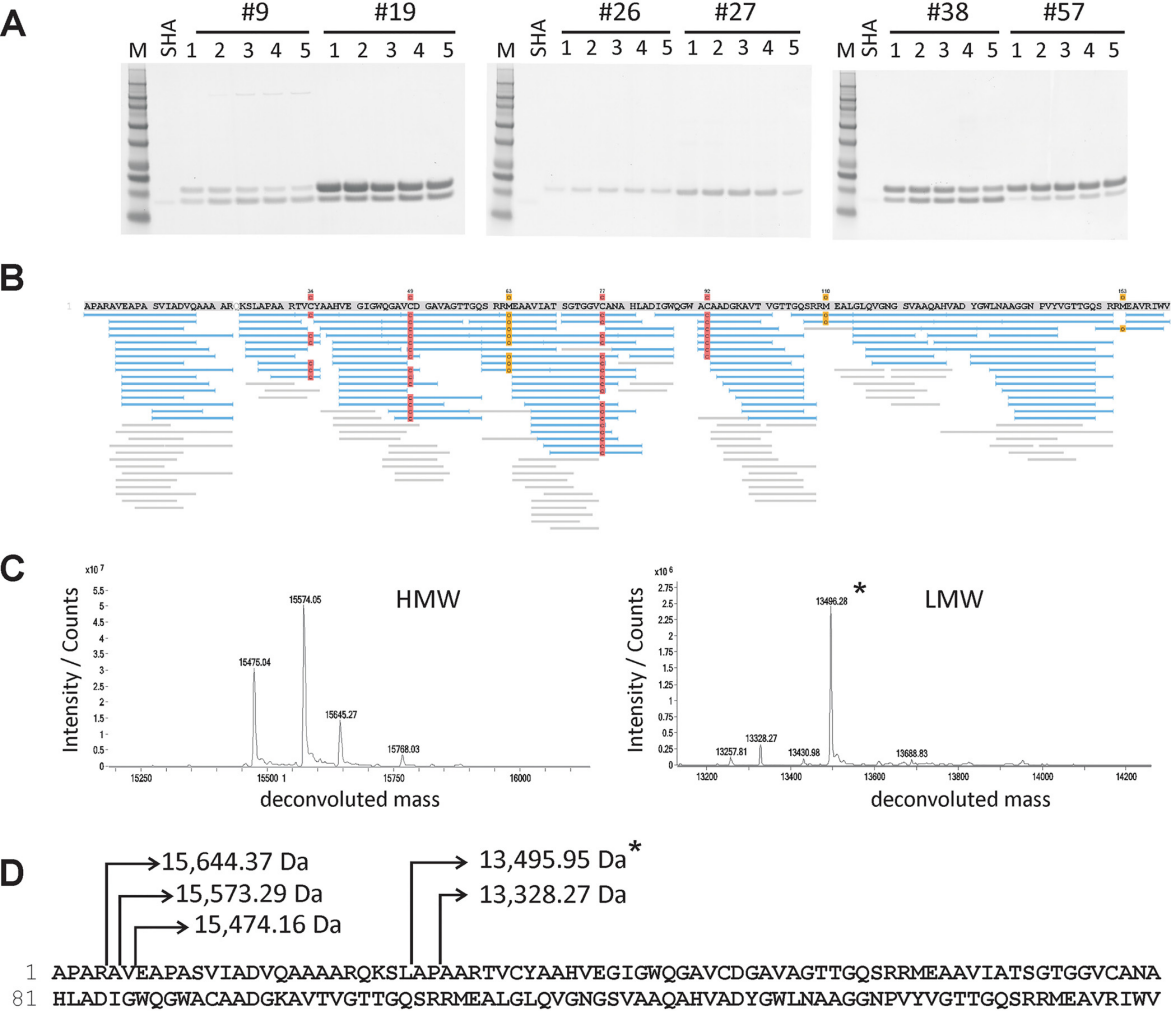
Purification and MS analysis of SHA homologues from culture supernatants of six IMC *Streptomyces* strains. (A) SDS-PAGE analysis of purified SHA homologues from six IMC strains, i.e., #9, #19, #26, #27, #38, and #57. Fifteen microliters of five fractions stepwise eluted from gum arabic gels for strain #9, #19, #26, #27, #38, and #57 SHA homologues and the authentic SHA (0.1 μg/lane) were loaded into wells. (B) LC-MS/MS analyses of tryptic and chymotryptic peptides aligned to the deduced amino acid sequence determined by DNA sequencing of the SHA gene in strain #27. Blue bars indicate database-matched peptides, and gray bars indicate *de novo* sequencing results. Carbamidomethylated cysteine residues are highlighted in red. (C) Deconvoluted electrospray ionization (ESI) Q-TOF MS spectra of high- and low-molecular-weight mass regions (HMW and LMW, respectively). (D) Pro-SHA protein #27 amino acid sequence with start sites indicated for #27 pro-SHA proteins and SHA proteins based on molecular masses determined by Q-TOF MS. The most abundant mature form is marked with an asterisk.

Protein purification was carried out using 2-liter culture supernatants of strains #9, #19, #26, #27, #38, and #57 by gum arabic affinity chromatography. SDS-PAGE of stepwise-eluted fractions of each purified SHA homologue, shown in Fig. 3A, verified the purity of the SHA homologues. Interestingly, two protein bands were observed in purified SHA homologues derived from strains #9, #19, #38, and #57. Their molecular weights (MWs) corresponded to apparently unprocessed (>15-kDa) and processed (∼13-kDa) proteins, and the latter MW was similar to that of authentic SHA.

The protein bands from each strain were cut out from the gels for tryptic and chymotryptic digestions. The resulting peptides were analyzed by LC coupled with high-resolution multistage mass spectrometry (MS/MS). Using this analysis, #9, #19, #27, #38, and #57 peptides were successfully matched to the respective amino acid sequences deduced from DNA sequences of corresponding PCR products, leading to determination of the amino acid sequences of the purified SHA analogues. The results of LC-MS/MS analysis of tryptic and chymotryptic peptides derived from the purified #27 SHA homologue are shown in Fig. 3B. The results for SHA homologues purified from four other strains, #9, #19, #38, and #57, are included in Fig. S1 in the supplemental material.

In addition to peptides matching the amino acid sequence of SHA homologues, as derived from DNA sequencing (Fig. 3B), intact quadrupole time-of-flight (Q-TOF) mass measurements of purified SHA homologues were successfully applied to match the full amino acid sequences of precursor and processed purified SHA analogues. This also revealed the location of proteolytic processing sites (Fig. 3C and D). When secreted, the signal peptide must have been removed to create the detected pro-SHA proteins with MWs of 15,644 to 15,474 Da. Further digestion of pro-SHA proteins at the N terminus of authentic SHA by associated proteases must occur to produce mature SHA proteins. Interestingly, the cleavage sites do not indicate apparent consensus sequences for yet-to-be-identified associated proteases, and some heterogeneous products have been detected.

In other words, as shown in Fig. 3B to D and in Fig. S1, mass spectrometric analyses of purified SHA homologues revealed that pro-SHAs start sites are located at −25, −24, and −23 amino acids from the N terminus of the authentic SHA, whereas the positions at −4 and −1 amino acids represent processing sites for mature SHA proteins (see Fig. 6C for details). The hypothetical protein in the genome of *S*. *lavendulae* contains 68 amino acids preceding the N terminus of the authentic SHA (5), and thus, 43 amino acids (i.e., 68 minus 25 amino acids) from the N terminus of the largest pro-SHA must be the length of the signal peptide or that of another type of sequence removed before secretion.

When tryptic and chymotryptic digests of the strain #26 SHA homologue were aligned with the amino acid sequence determined by DNA sequencing, it was found that two sequences consisting of 3 amino acids each were not covered by those peptides. Since this result was most likely due to the low abundance of the #26 SHA homologue used in the original analysis (Fig. 3A), an additional experiment was carried out. Using different enzyme digestions on increased amounts of #26 SHA proteins as described in Materials and Methods, we were able to match LC-MS/MS data to peptides that cover the entire amino acid sequence of the #26 SHA homologue. In addition, we confirmed that the purified *S. lavendulae* SHA (*Lav*) produced peptides which aligned to the CCM 3239 genome-derived amino acid sequence.

### Expression levels of the SHA homologues were estimated based on the yield of pure SHA homologue proteins.

Table 3 summarizes the amounts of SHA homologues purified from culture supernatants of the six IMC strains we have chosen. Yields of the SHA homologues were, however, significantly lower than that of the authentic SHA purified from *Streptomyces* sp. strain 27S5 from previously reported data (3). Yields of the best SHA homologue producers, strains #19, #38, and #57, ranged at best from 21% to 26% of those of the lost strain, *Streptomyces* sp. strain 27S5. Note that Table 2 also includes a summary of the SHA homologue purification from the culture supernatant from *S. lavendulae* (*Lav*), our originally used *S. lavendulae* strain (ATCC 14158) from which rSHA was constructed (5). Purification of SHA homologues from *S. lavendulae* and *Streptomyces* sp. Mg1 is presented in the following section.

**TABLE 3:** Summary of purification

SHA source	Total SHA homologue purified (mg)/2 liters of culture supernatant	Level of production relative to original SHA (%)
Original SHA^*a*^	8.0	100
Strains		
#9	0.590	7.3
#19	2.08	26.0
#26	0.412	5.2
#27	0.655	8.2
*Lav*	1.16 (0.050 mg from 86 ml)	14.5
#38	1.693	21.2
#57	2.09	26.1

a The yield of the original SHA obtained from *Streptomyces* sp. 27S5 was 60 mg from 15 liters, equivalent to 8 mg from 2 liters (3).

### Expression and purification of SHA homologues from *S. lavendulae* and *Streptomyces* sp. Mg1.

*S. lavendulae* (*Lav*) and *Streptomyces* sp. Mg1 were grown under the same culture conditions as the other six strains, except that they were cultured in one flask each. WB of the culture supernatants which were concentrated 4-fold by TCA precipitation is shown in Fig. 2, labeled *Lav* and Mg1, respectively. The results confirmed that both strains produced and secreted pro-SHA proteins in culture broths. *Streptomyces* sp. Mg1 produced a pro-SHA with a lower MW than other pro-SHA proteins, whereas *S. lavendulae* secreted a pro-SHA with a MW similar to those from the other 9 strains (Fig. 2).

**FIG 4:**
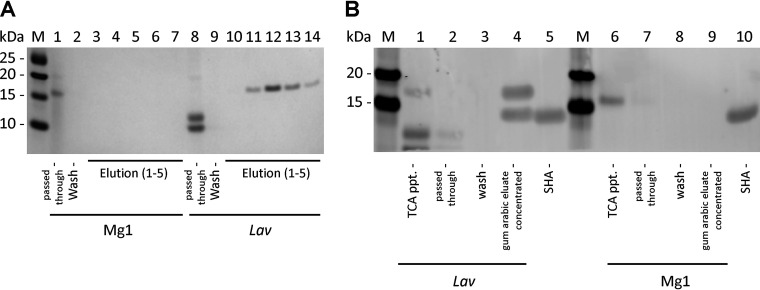
Purification of SHA homologues by gum arabic affinity chromatography from culture supernatants of *Streptomyces* sp. Mg1 and *S. lavendulae* (*Lav*; ATCC 14158). (A) Coomassie blue-stained SDS-PAGE gel of purified *Lav* protein bands (lanes 11 to 14). Affinity column purification of *Streptomyces* sp. Mg1 homologue resulted in no bands in the eluate from the column (lanes 3 to 7). (B) Western blots of the SDS-PAGE gel confirming the presence of the SHA homologues in the *Lav* eluate (lane 4) but not in the *Streptomyces* sp. Mg1 eluate (lane 9). The gum arabic eluates seen in panel A were combined and concentrated 10-fold for immunoblotting. ppt., precipitate.

Figure 4A demonstrates SHA homologue proteins bound to and eluted from the gum arabic column (lanes 11 to 14). WB shown in Fig. 4B revealed that although the culture supernatant contained pro-SHA and two small cross-reactive proteins in lane 1, the purified fraction in lane 4 contained two bands, presumably the pro-SHA and its processed SHA. In contrast, the pro-SHA protein of *Streptomyces* sp. Mg1 apparently passed through the column, since no protein eluted from the affinity column (Fig. 4A, lanes 3 to 7; Fig. 4B, lane 9). The *Streptomyces* sp. Mg1 pro-SHA is clearly detected by WB in the concentrated culture supernatant (Fig. 4B, lane 6). It is invisible in the pass-through (lane 7), the wash fractions (lanes 8), and most importantly in the 10-fold-concentrated eluates (lane 9). These results revealed that *Streptomyces* sp. Mg1 produced an anti-SHA cross-reactive protein which is most likely the hypothetical SHA homologue that was identified in its genome in 2014 (5). However, this Mg1-derived SHA homologue cannot bind to Rha/Gal residues of gum arabic gels, indicating that it lacks this specific carbohydrate binding activity.

### HA activities of purified SHA homologues.

Purified SHA homologues of strains #9, #19, and #38 exhibited high HA activities to type B erythrocytes in the order #9 ≈ #19 > #38 but not to type A or O erythrocytes (Table 4), confirming the same type B specificity as that of the authentic SHA. The SHA homologue of #57 showed significant HA activity. SHA homologues of #26 and #27, however, did not hemagglutinate at all. The lack of HA activity in #26 and #27 SHA homologues is apparently associated with a lack of the processed SHA proteins, as shown in Fig. 3A. This observation that pro-SHA proteins do not have HA activity strongly indicates that a second carbohydrate binding site is not available to agglutinate erythrocytes, suggesting that pro-SHAs are monovalent. Unlike many lectins known to consist of subunits, each of which may contain one binding site, SHA is a monomer of 13 kDa that possesses two binding sites based on analytical ultracentrifuge analysis (6) and Scatchard’s plot analysis (4), respectively.

**TABLE 4:** HA activities of purified SHA homologues

Source of purified SHA	Expt 1 HA titer in indicated blood type			Expt 2 HA titer in blood type B
	B	A	O	
Original SHA	64	0	0	64
Strains				
#9	64	0	0	64
#19	128	0	0	64
#26	0	0	0	0
#27	0	0	0	0
#38	32	0	0	16
#57	1	0	0	4

Table 5 summarizes the binding properties of SHA homologues and also the binding activities of chemically modified SHA and rSHA, which were conjugated at the N terminus with a green fluorescent protein (GFP) or modified with smaller molecules such as biotin. GFP-conjugated rSHA is similar to pro-SHAs, since both contain N-terminal domains preceding the SHA domains. The above-mentioned conclusion that N-terminal domains existing in pro-SHA proteins appear to inhibit HA activity is consistent with the observation that the presence of large proteins such as GFP attached to the N terminus of rSHA inhibited HA activity. These results imply that the extra N-terminal domains hinder one binding site, resulting in only one other binding site available for the pro-SHA proteins as well as the GFP-rSHA conjugate. In contrast, modification of SHA with a small molecule such as biotin did not affect HA activity.

**TABLE 5:** Binding activities of purified SHA analogues and modified SHA and rSHA proteins^*a*^

Protein	Binding to gum arabic gells	Binding to glycans	HA activity
SHA analogue from *Streptomyces* strain			
#9	+	ND	+
#19	+	ND	+
#26	+	ND	−
#27	+	ND	−
*Lav*	+	ND	−
#38	+	ND	+
57	+	ND	±
Mg1	−	ND	ND
			
Modified rSHA or SHA			
GFP-rSHA	+	Cell surface	−
Biotinylated rSHA	+	Glycan array	ND
Biotinylated SHA	+	Glycan array	+
Authentic SHA	+	ND	+

a ND, not determined; Cell surface, bound to bacterial cells (5); Glycan array, specifically bound to glycan array as published previously (5).

### Assessment of specific HA activities of processed SHA homologues.

As observed clearly in the purified SHA protein homologues (Fig. 3A, #9, #19, #38, and #57), pro-SHA proteins were apparently digested by yet-to-be-identified proteases during and after purification. This suggests that proteases are also secreted from those *Streptomyces* strains producing SHA homologues.

**FIG 5:**
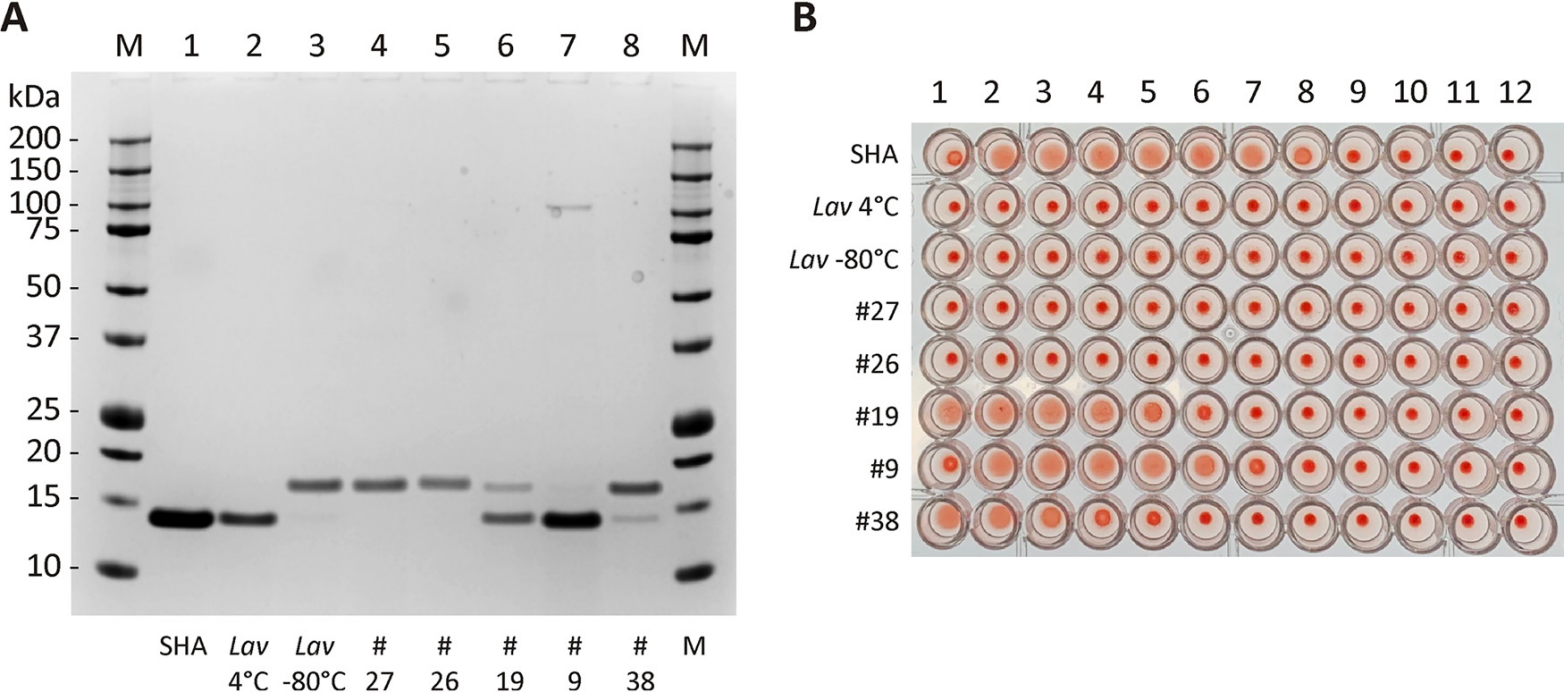
Determination of specific HA activities of purified SHA homologues. (A) Coomassie blue-stained SDS-PAGE gel of various batches of purified SHA homologues as labeled. (B) The same samples shown in panel A were assayed for their HA activities. Shown here is one of the duplicate HA assays from Table 6.

We found that the purified pro-SHA of *S. lavendulae* (*Lav*), which is the same SHA homologue produced by #27, was extremely susceptible to processing by associated proteases. We thus attempted to produce the SHA domain by incubating the purified *Lav* SHA homologue at 4°C for 4 weeks, which resulted in “processed SHA” (Fig. 5A, lane 2), whereas the purified *Lav* SHA homologue kept at −80°C remained as an intact pro-SHA protein (Fig. 5A, lane 3). These two *Lav* samples together with 5 other purified samples containing pro-SHAs (#26 and #27) and both pro-SHAs and processed SHAs (#19, #9, and #38) were subjected to SDS-PAGE for protein estimation (Fig. 5A) and HA assays (Fig. 5B; Table 4, experiment 1) in parallel. The results clearly support the finding that *Lav* at 4°C, *Lav* at −80°C, #27, and #26 did not exhibit HA activity (Fig. 5B). In contrast, SHA homologues containing processed SHA proteins (#19, #9, and #38) showed high HA activity. The SHA homologue of strain #9, consisting of mostly the processed form, exhibited the highest HA activity among the three. However, *Lav* at 4°C, which hypothetically contained the processed SHA homologue, did not show evidence of HA activity. The reason why *Lav* at 4°C did not show HA activity requires further investigation.

Based on the amount of processed SHA proteins in the samples used for HA assays and the results of duplicate HA assays, specific activities of authentic SHA and SHA from strains #19, #9, and #38 were estimated (Table 6). The data indicate strong specific HA activities of SHA proteins from #19, #9, and #38 and support the finding that the processed SHA homologues have two active carbohydrate binding sites, which are required for hemagglutination.

**TABLE 6:** Comparison of specific HA activities of purified SHA homologues to that of an authentic SHA

Protein bands corresponding to SHA and its homologs	Expt 1 titer	Expt 2 titer	Titer avg	Relative SHA protein amount (%)	Sp act
SHA	256	128	192	100	192
*Lav* at 4°C	0	0	0	36	0
*Lav* at −80°C^*a*^	0	0	0	27.3	0
#27	0	0	0	25.7	0
#26	0	0	0	19.3	0
#19 top^*a*^	16	16	16	6.2	0^*b*^
#19 bottom				21.3	75
#9	64	64	64	62.1	121
#38 top^*a*^	8	8	8	24.4	0^*b*^
#38 bottom				3.7	216

a Pro-SHA protein.

b Pro-SHA proteins are assumed to have no HA activity (see the text).

### Comparison of amino acid sequences of 10 IMC-originated SHA homologues with that of authentic SHA.

**FIG 6:**
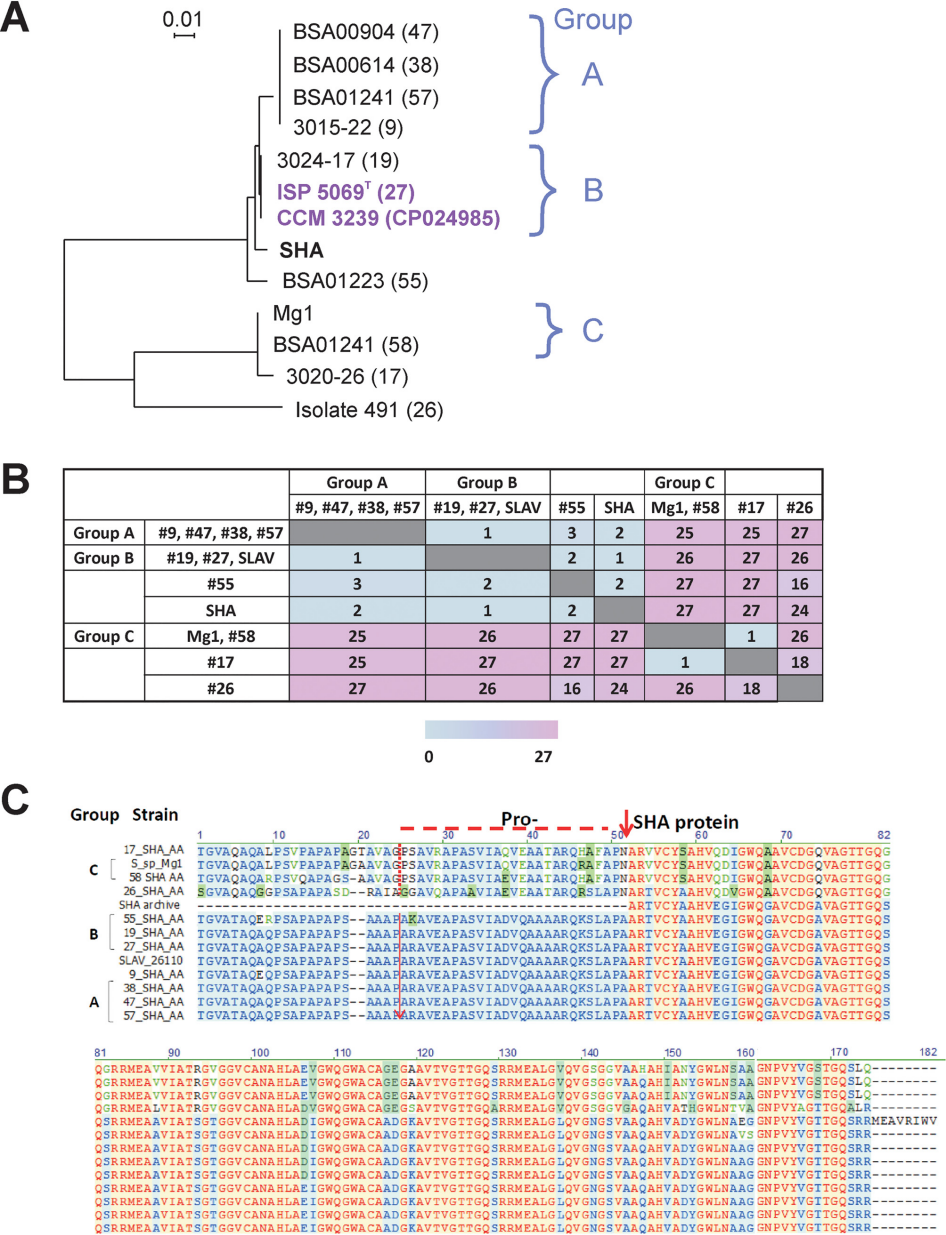
Comparison of amino acid sequences of SHA homologues derived from 10 IMC strains together with SLAV (*S. lavendulae* subtype CCM 3239 [7]) and *Streptomyces* sp. Mg1. (A) Phylogenetic tree of SHA domains of the SHA analogues (SHA proteins) and the authentic SHA. Within each group, A, B, and C, the SHA amino acid sequences are identical. *S. lavendulae* strains ISP 5069^T^ (#27) and CCM 3239 (accession no. CP024985) are in magenta. (B) Heat map of variation between SHA homologues as shown by the number of amino acid differences (1 to 27, highlighted in blue to pink). (C) Alignment of amino acid sequences of SHA and its homologues. The N terminus of SHA proteins (red arrow) and the start site of pro-SHA proteins (dashed red lines) are indicated. The color scheme shows similarities, from most similar (red) to less similar (blue) and least similar (green and black).

In addition to the six strains chosen for purification of SHA homologues, four more strains from the IMC collection, #17, #47, #55, and #58, were included in this study, as summarized in Table 2. Sequencing of the SHA homologues of 10 strains provided us with amino acid sequence information for their SHA homologues. Phylogenetic tree analysis of SHA domains from those 10 strains as well as *S. lavendulae* CCM 3239 (7) (abbreviated SLAV), the authentic SHA (5), and that from *Streptomyces* sp. Mg1 is shown in Fig. 6A. The SHA homologues having the same sequences are categorized into group A (#9, #47, #38, #57), group B (#19, #27, SLAV), and group C (Mg1, #58). A heat map of the differences in amino acid sequences among SHA proteins is shown in Fig. 6B. SHA homologues closely related to the authentic SHA are those in group A (#9, #47, #38, #57) and group B (#19, #27, SLAV) and that in strain #55, shown in blue, whereas group C (Mg1, #58) and strain #17 and #26 SHA homologues are quite different from the authentic SHA, as shown in pink. Of the 10 SHA homologues, the SHA homologue of strain #26, in particular, appeared to be most distant from the rest of the SHAs. In fact, we found that PCR primers which amplified other SHA homologue regions did not work for the strain #26 genome, so that specially designed primers had to be used to amplify the #26 homologue (Table 1).

The amino acid sequences of all SHA homologues listed in Fig. 6A and B are shown in Fig. 6C in comparison to that of the authentic SHA. Amino acid sequences of 10 IMC strains, marked from positions 1 to 175, were deduced from DNA sequences obtained by PCR using primers listed in Table 1. In addition to the 10 IMC strains, those of *Streptomyces* sp. Mg1 and SLAV (7) are also aligned. The authentic SHA sequence lacking amino acids prior to its N terminus is aligned, which indicates potential N termini of SHA proteins in pro-SHA proteins (Fig. 6C). The start sites of both pro-SHA and SHA proteins are indicated in Fig. 6C.

Group A SHA homologues of strains #9, #38, and #57 share the same amino acid sequence, but interestingly, the SHA homologue of #9 had the best hemagglutination activity, followed by the #38 homologue. The SHA homologue purified from #57 showed much lower HA activity (Table 4). The ratios of high- and low-MW bands of the three purified SHA homologues were roughly 1:10 for #9 and #38 and 20:1 for #57 (data not shown). The observed difference in HA activity must be attributed to the presence of processing enzymes in culture supernatants and purified samples, which convert pro-SHAs to SHA proteins. Low HA activity in the purified strain #57 SHA homologue may suggest the possibility either that #57 does not express such proteases or that it may secrete proteases with much lower activities than those secreted from strains #9 and #38.

### Comparison of 16S rRNA and SHA homologue phylogeny.

**FIG 7:**
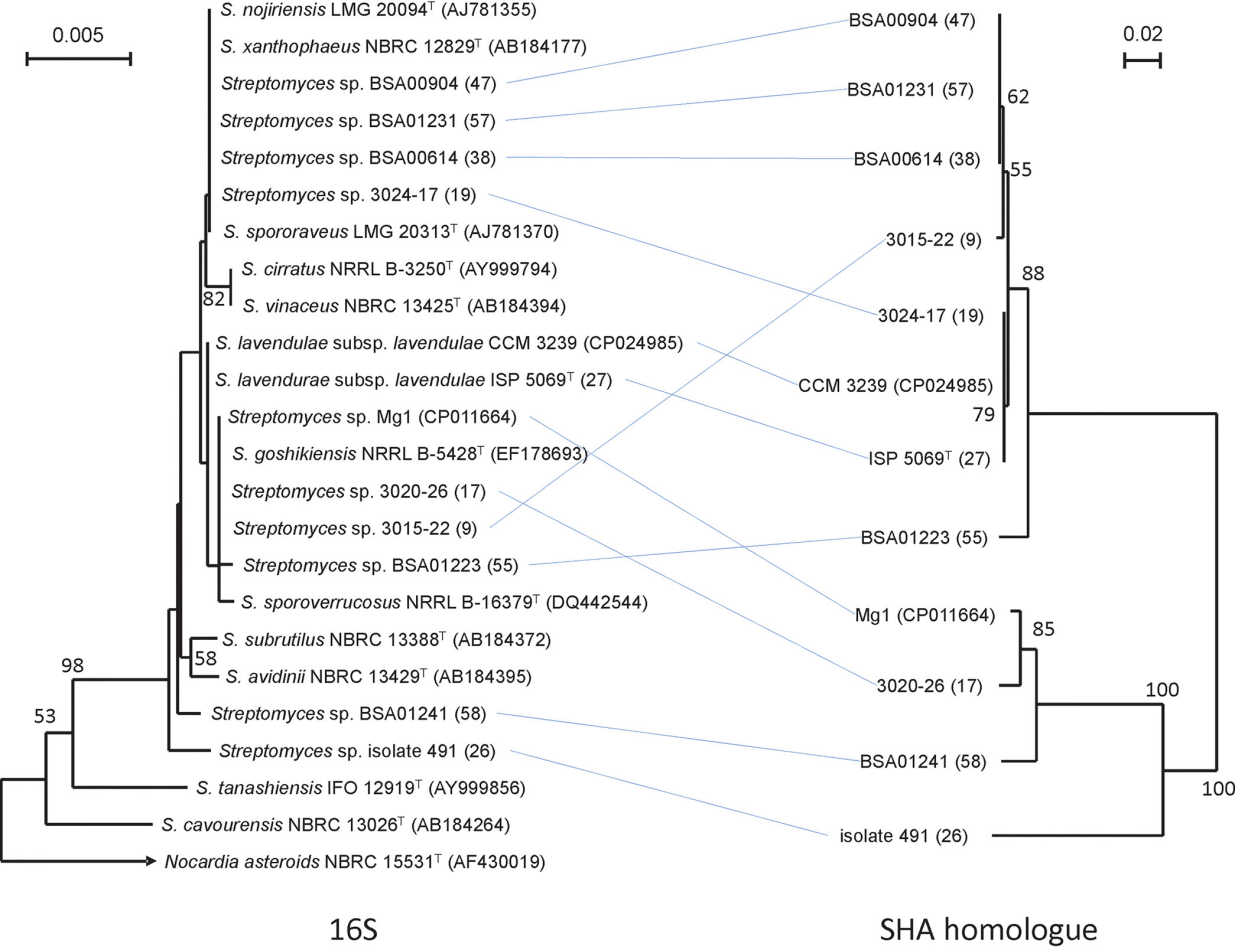
Relationship of the 16S rRNA phylogenetic tree to that of SHA homologues (pro-SHA proteins). The phylogenetic tree of pro-SHA, which is nearly the same as the one in Fig. 6A, except that the authentic SHA is not included, is on the right. Ten pro-SHA proteins and those of *S. lavendulae* CCM 3239 (7) and *Streptomyces* sp. Mg1 are connected to corresponding positions in the phylogenetic tree of 16S rRNA on the left. Information about the *Streptomyces* strains shown here is given in Table 7.

When comparing the phylogenetic tree of 16S rRNA and that of pro-SHA homologues from 10 IMC strains as well as *S. lavendulae* (SLAV) and *Streptomyces* sp. Mg1 side by side, the phylogenetic relationship between the SHA gene homologue and the 16S rRNA gene is largely consistent. However, some inconsistencies are obvious, with a mosaic pattern of distribution of the SHA gene revealed in the *S. lavendulae*-related strains with 99.6% to 100% homology to 16S rRNA of *S. lavendulae* (Fig. 7; Table 7). This cannot be explained by multiple deletions of a gene inherited from a common ancestor, suggesting that deletion and horizontal transfer may have occurred in this phylogenetic group.

**TABLE 7:** Information about *Streptomyces* strains, including 10 IMC strains and other closely related strains shown in Fig. 7

Strain	IMC designation	Accession no. of 16S rRNA	Accession no. of SHA homologue	Locality of source	Source	% Homology to CCM 3239
*Streptomyces* sp. 3015-22	#9	LC600142	LC600152	Aomori, Japan	Soil	100
*Streptomyces* sp. 3020-26	#17	LC600143	LC600153	Japan	Soil	100
*Streptomyces* sp. 3024-17	#19	LC600144	LC600154	Nagano, Japan	Soil	99.9
*Streptomyces* sp. isolate 491	#26	LC600145	LC600155	Tokyo, Japan	Leaf litter	99.5
Streptomyces lavendulae subsp. *lavendulae* ISP 5069^T^	#27	LC600146	LC600156	NA (type strain)		100
*Streptomyces* sp. BSA00614	#38	LC600147	LC600157	Japan	Soil	99.9
*Streptomyces* sp. BSA00904	#47	LC600148	LC600158	Japan	Soil	99.9
*Streptomyces* sp. BSA01223	#55	LC600149	LC600159	Japan	Soil	99.9
*Streptomyces* sp. BSA01231	#57	LC600150	LC600160	Japan	Soil	99.9
*Streptomyces* sp. BSA01241	#58	LC600151	LC600161	Japan	Soil	99.7
Streptomyces avidinii NBRC 13429^T^		AB184395			Soil	99.7
Streptomyces cavourensis NBRC 13026^T^		AB184264			Soil	98.4
Streptomyces cirratus NRRL B-3250^T^		AY999794				99.8
Streptomyces goshikiensis NRRL B-5428^T^		EF178693		Fukushima, Japan	Soil	99.9
Streptomyces lavendulae subsp. *lavendulae* CCM 3239		CP024985	CP024985			
Streptomyces nojiriensis LMG 20094^T^		AJ781355		Hokkaido, Japan	Soil	99.9
*Streptomyces* sp. Mg1		CP011664	CP011664		Soil	99.6
Streptomyces spororaveus LMG 20313^T^		AJ781370			Soil	99.9
Streptomyces sporoverrucosus NRRL B-16379^T^		DQ442544			Soil	99.7
Streptomyces subrutilus NBRC 13388^T^		AB184372		Aomori, Japan	Soil	99.7
Streptomyces tanashiensis IFO 12919^T^		AY999856		Tokyo, Japan	Soil	98.8
Streptomyces vinaceus NBRC 13425^T^		AB184394				99.8
Streptomyces xanthophaeus NBRC 12829^T^		AB184177			Soil	99.9
Nocardia asteroides NBRC 15531^T^		AF430019				89.2

### Database search for SHA homologues results in identification of SHA genes in syntenic regions.

**FIG 8:**
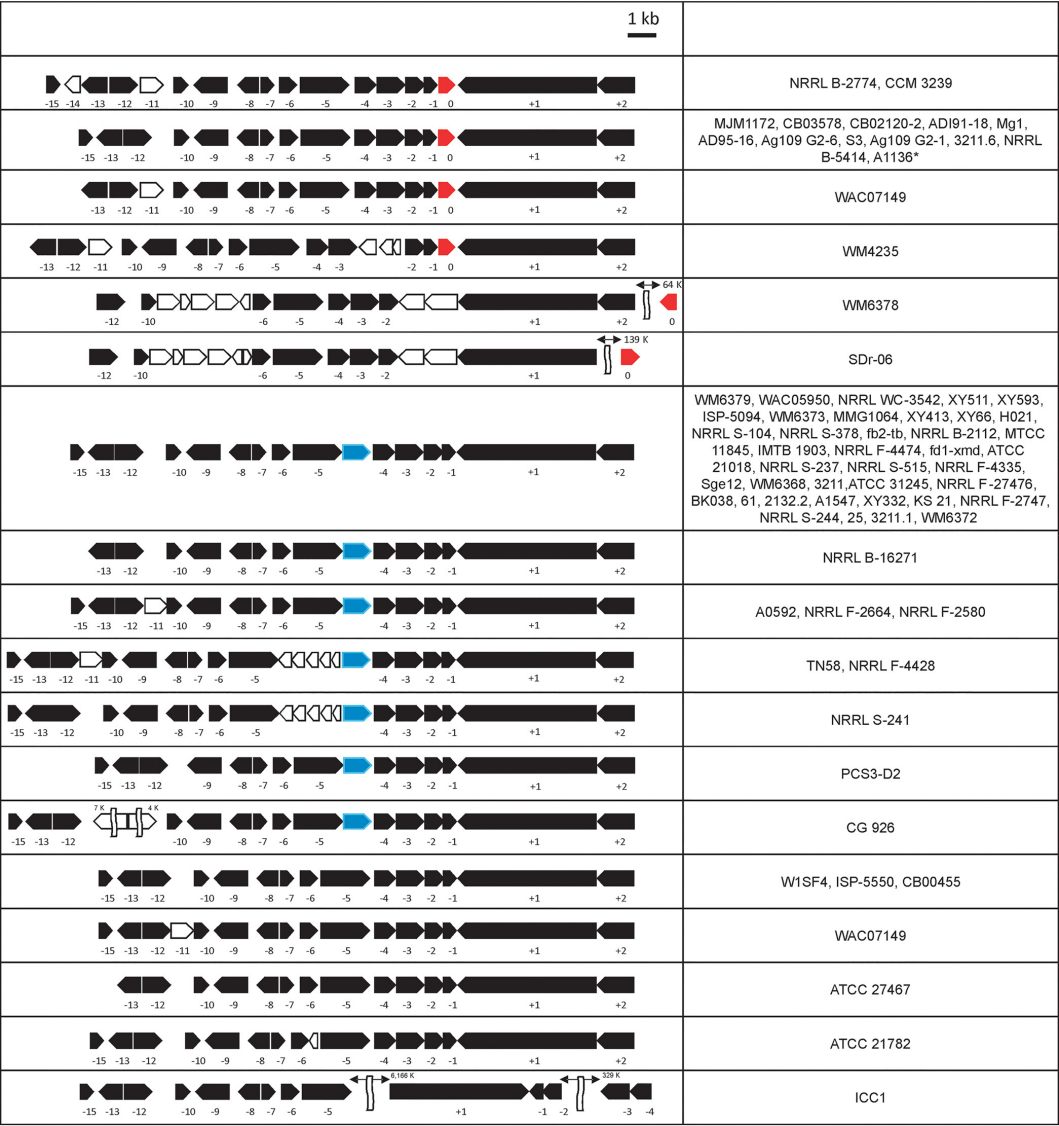
SHA locus. Syntenic regions carrying the SHA gene (red shape, 0) in multiple strains listed in Table 8 are shown. The SHA gene is located in syntenic regions consisting of ORF −15 to ORF +2, as seen in Streptomyces lavendulae subsp. *lavendulae* CCM 3239 at the top of the figure. Two strains with SHA genes outside the syntenic region (WM6378 and SDr-06) are listed underneath the syntenic regions with SHA as the reference. The listed syntenic regions (Table 8) are from groups a and b, 16 strains with the SHA gene, group d1, 51 strains with LP (blue shape, between ORFs −4 and −5), and group d2, 7 strains without LP. Similar syntenic regions carry either the SHA or LP gene or lack SHA and LP genes altogether.

To study the distribution of the SHA gene homologue in *S. lavendulae*-related strains, an entirely different approach from the above-described protein-focused one was taken. That is, the entire genome sequences of 1,234 *Streptomyces* strains available were downloaded from the NCBI genome databases and constructed into a local BLAST database. A homology search for the SHA gene revealed 18 *Streptomyces* strains carrying an SHA gene. They are categorized as groups a (12 strains), b (4 strains), and c (2 strains) according to the homology values to *S. lavendulae*, as summarized in Table 8. Interestingly, group a and b strains carried the SHA gene in a syntenic region, as illustrated in Fig. 8. The synteny region consists of 12 to 13 genes upstream, ORFs −1 to −15, excluding −11 and −14 and some also lacking −15, and 2 genes downstream, ORF +1 and ORF +2. The numbers were assigned to the ORFs of this synteny region of *S. lavendulae* CCM 3239 with the SHA gene used as the reference, i.e., ORF 0 (Fig. 8). The flanking region of the SHA genes in the genomes of the remaining two strains did not show syntenicity, and the 16S rRNA genes were also phylogenetically distant from that of *S. lavendulae*.

**TABLE 8:** Results of BLAST search for SHA homologues from 1,234 *Streptomyces* strains and phylogenetically closely related strains

Group	Category description	SHA gene found	Strains^*a*^
a	12 strains with >99% 16 rRNA homology to CCM 3239 (*S. lavendulae*)	Yes (in synteny)	ADI95-16, CB02120-2, CB03578, AD191-18, MJM1172, Mg1, NRRL B-2774, CCM 3239, WM4235, NRRL B-5414, A1136, WAC07149
b	4 strains with ∼98 to 99% 16 rRNA homology to CCM 3239 (*S. lavendulae*)	Yes (in synteny)	Ag109 G2-6, S3, Ag109 G2-1, 3211.6
c	2 strains with ∼98% 16 rRNA homology to CCM 3239 (*S. lavendulae*)	Yes (no synteny)	WM6378, SDr-06
d1	Strains with >99% 16 rRNA homology to CCM 3239 (*S. lavendulae*)	No (syntenic region similar to those of group a and b strains but lacks SHA gene)	With LP: IGB124, WACO5950, TN58, NRRL F-4428, MTCC 11845, IMTB 1903, NRRL F-4474, fd1-xmd, A0592, PCS53-D2, NRRL F-2664, ATCC21018, CG 926, WM6368, 3211, Sge12, NRRL F-2580, A1547, KS 21, NRRL B-16271, NRRL F-2747, ATCC27476, BK038
d2			Without LP: CB00455, ATCC 27467, ATCC 21782, NRRL ISP-5550, WAC07061, W1SF4

a A list of the full name, accession number, and percent homology to *S. lavendulae* 16S RNA of the strains is given in Table S2 in the supplemental material. The syntenic regions of those strains are shown in Fig. 8.

**FIG 9:**
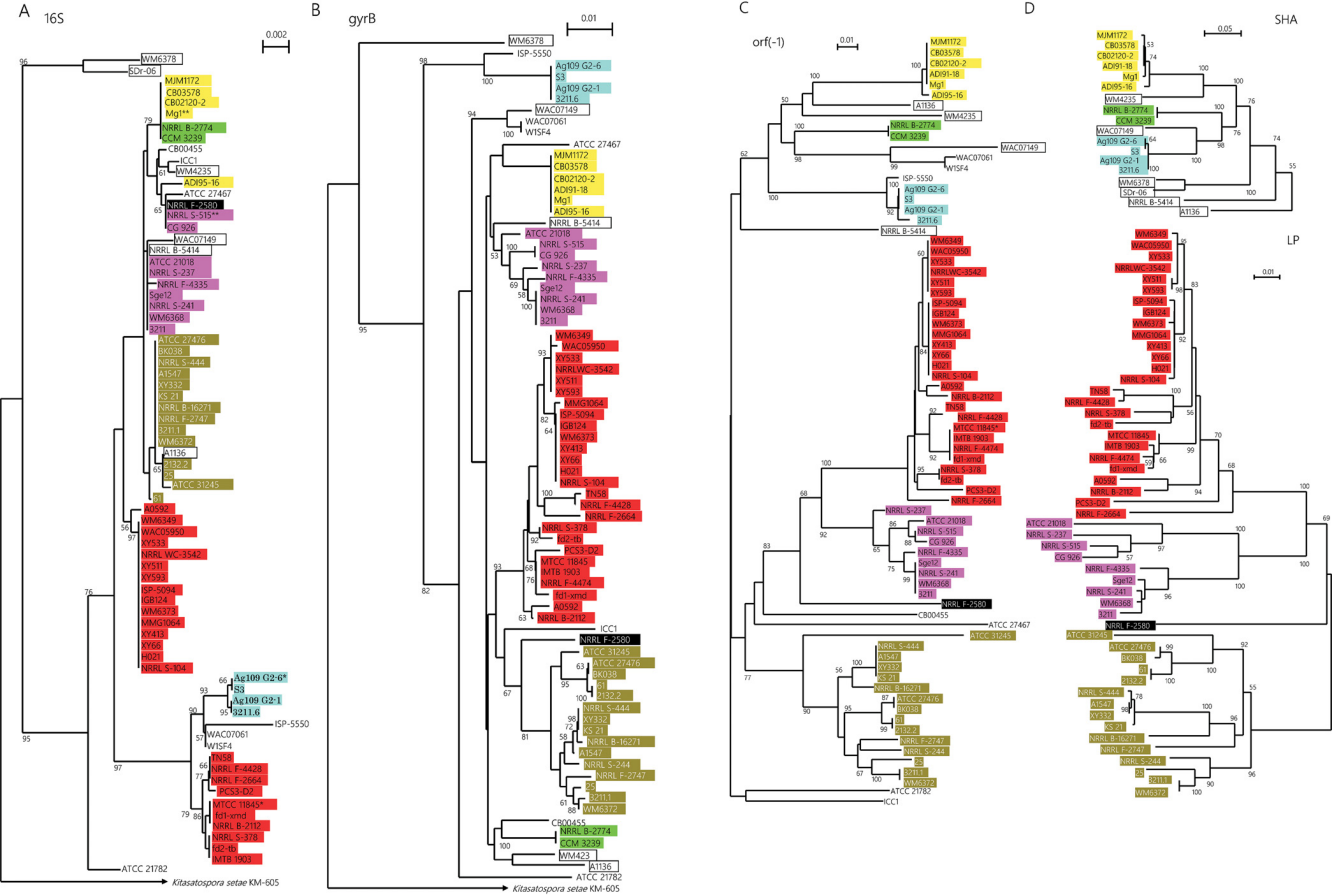
Phylogenetic trees of 16S rRNA (A), *gyrB* (B), ORF −1 (C), and database-derived SHA or LP genes (D) among *S. lavendulae*-related strains listed in Table 8. A BLAST search for SHA homologues from 1,234 *Streptomyces* strains resulted in the identification of 18 homologues (Table 8, groups a, b, and c) whose phylogenetic relationships are shown in panel D. The SHA gene in 16 strains from group a and b was found in the syntenic regions, whereas two SHA homologues in group c were encoded outside the syteny region (WM6378, SDr-06). The syntenic region carrying SHA gene 0 in 16 *Streptomyces* strains and very similar syntenic regions which contain the LP gene instead of the SHA gene (Table 8, group d1) and ones without the LP or SHA gene (Table 8, group d2) are shown. Those strains contain ORF −1, whose phylogenetic tree (C) is obviously in good agreement with the phylogenetic tree shown in panel D. The tree with the 18 SHA homologues, shown in the upper portion of panel D, is constructed from 3 clades (highlighted in yellow, lime, and cyan) and 6 singletons. The bottom portion of panel D demonstrates the phylogenetic relationships of the 51 LP genes identified. It consists of 3 clades (highlighted in red, magenta, and olive) and one singleton (highlighted in black).

We found that strains closely related to *S. lavendulae* but without SHA also share the same syntenic region (group d), as shown in Fig. 8. Many of them share a lipoprotein (LP) gene upstream of ORF −4 (group d1). The top portion of Fig. 9D demonstrates phylogenetic relationships of the 18 SHA homologues. The tree is constructed with 3 clades (highlighted in yellow, lime, and cyan) and 6 singletons. The bottom portion of Fig. 9D demonstrates the phylogenetic relationship of the 51 LP genes identified. It consists of 3 clades (highlighted in red, magenta, and olive) and one singleton (highlighted in black). If the SHA gene was transferred vertically from a common ancestor to these strains, they should be monophyletic in the 16S rRNA phylogenetic tree. However, the cyan-highlighted clade, as well as the two singletons (WM6378, SDr-06) that are phylogenetically distant from *S. lavendulae*, do not show monophyly with other SHA-possessing strains on the 16S rRNA gene tree (Fig. 9A). In addition, the yellow-highlighted clade has lost its monophyly on the 16S rRNA gene tree. The LP-bearing strains also show a similar phenomenon. Furthermore, although the SHA and LP gene-possessing strains show exclusivity, they show a mosaic distribution on the 16S rRNA gene tree. Mosaic distributions of SHA gene-possessing strains were also observed on the *gyrB* (Fig. 9B) and *rpoB* (data not shown) trees.

To understand why the SHA gene is frequently associated with *S. lavendulae*-related strains, and shows such a phylogenetically mosaic distribution, we took advantage of genomic information of strains listed in Table 8 for a close investigation of the SHA gene locus. As described above, most SHA genes locate within a syntenic region. The syntenic region is specific to *S. lavendulae*-related strains, and the syntenicity is lost as the phylogenetic distance from *S. lavendulae* increases (data not shown). The phylogenetic tree of ORF −1 is in excellent agreement with the phylogenetic trees of SHA and LP (Fig. 9C and D).

## DISCUSSION

The goal of this study was to recapture the lost strain, if possible, or at least to find similar strains producing SHA homologues so we could investigate how SHA proteins are biosynthesized and what the biological roles of SHA may be. When the primary structure of SHA was to be determined in April 2014, we luckily identified one homologue in the genome of *Streptomyces* sp. Mg1 deposited in the database just 8 months prior to our database search. Then, 2 months later, we were extremely fortunate to find the *S. lavendulae* SHA homologue, which must have been deposited around the same time. As we published previously (5), the *S. lavendulae* SHA homologue domain differs by only one amino acid from the authentic SHA, so we were able to produce recombinant SHA protein, which showed the same glycan specificity as that of the authentic SHA. While preparing our publication in 2016, we found 6 additional *Streptomyces* strains encoding SHA homologues with amino acid sequence identities ranging from 55% to 80% (see Fig. 4 in reference 5). In late 2019, when a renewed database search was carried out, we found 18 *Streptomyces* strains encoding SHA homologues, of which 13 strains were newly acknowledged.

Since we could not detect the expression of SHA homologue proteins by HA assays in culture supernatants of *S. lavendulae* and *Streptomyces* sp. Mg1, both of which encoded SHA homologue hypothetical proteins, we were not certain whether or not SHA proteins were expressed and secreted from those bacterial strains. The present study revealed that the *S. lavendulae* SHA homologue (*Lav*) is secreted as a pro-SHA that does not have divalent binding sites, whereas *Streptomyces* sp. Mg1 expressed the SHA homologue-like protein which entirely lacks carbohydrate binding activities. This fact clearly indicates that previously used HA assays could not have detected the presence of an SHA homologue in their culture supernatants. In the present study, we prepared and used rabbit anti-SHA sera, which confirmed the expression and secretion of SHA homologues from both strains. The unexpected result from purification experiments was that the hypothetical SHA homologue protein from *Streptomyces* sp. Mg1 did not bind to gum arabic gels, which suggested that it lacks l-Rha/d-Gal binding activity. In contrast, the SHA homologue from the *S. lavendulae* strain we purchased from ATCC (*Lav*) was purified and determined to have the same sequence as IMC strain #27 (purified and sequenced in this study) as well as CCM 3239 (database derived) (7).

It appears that two strains, #26 and #27, lacked such a processing mechanism, since the #26 and #27 SHA homologues remained in the pro-SHA form. Pro-SHAs were presumably monovalent, since they do not hemagglutinate type B erythrocytes but were able to bind to gum arabic gels, which resulted in the successful purification of the seven pro-SHAs. It should be noted, however, that strain #27 was *S. lavendulae* ISP 5069 (=ATCC 14158), which had been kept at IMC, while the strain we previously studied (5) was *S. lavendulae* (*Lav*) purchased from the ATCC (ATCC 14158). Peptide alignments to the deduced SHA homologue proteins of the two strains revealed the same amino acid sequence of their pro-SHA proteins, as expected. Unexpectedly, however, we found a very striking difference between the two purified SHA homologues. That is, strain #27 pro-SHA was hardly processed to mature SHA, whereas *Lav* pro-SHA protein was much more susceptible to processing. This could be explained by the difference in the associated proteases’ activities and/or secretion levels between strains #27 and *Lav*. We intentionally produced the processed SHA from *Lav* pro-SHA in the hope of demonstrating that processing pro-SHA to SHA protein could result in the appearance of HA activity, which pro-SHA proteins lack. Unexpectedly, as shown in Fig. 5, the processed *Lav* SHA, which was assumed to be equivalent to the SHA protein, did not show HA activity. Q-TOF MS analyses indicated that #9, #19, and #38 SHA homologues appear to have been processed to the N terminus of the authentic SHA, whereas *Lav* SHA processing seems to end at −3, leaving the APA sequence just prior to ARTV, which is the N-terminal sequence of the authentic SHA (Fig. 3C and D; see Fig. S1 in the supplemental material). Further investigation is required to understand the potential effects of these extra amino acids on HA activity.

This study effectively identified *Streptomyces* strains producing SHA homologues. However, it clearly demonstrated that since the WB results indicated that fully processed SHAs were hardly detected in culture supernatants of those strains, and since pro-SHA proteins cannot hemagglutinate type B, the original screening protocol, which successfully identified the authentic SHA (1, 2), is not sensitive enough to detect the presence of type B-specific hemagglutinins in culture broths of those 7 strains. In other words, *Streptomyces* sp. 27S5 secreted a completely processed SHA at a much higher level than those pro-SHAs from the 7 strains. When SHA was originally screened by HA assays, the titer was 2 to 4 (1, 2), which means that even though those 7 strains produced SHA homologues in fully HA active SHA forms, HA assays would only give a titer of 1, since protein secretion levels of the highest producers are one-fourth that of *Streptomyces* sp. 27S5. A titer of 1 is not considered to be positive for HA assays. In reality, since those culture supernatants mainly contained pro-SHAs, the detection of pro-SHAs by HA assays would not have been possible, because monovalent pro-SHA cannot exhibit HA activity, as it requires bivalent SHA. Furthermore, although we succeeded in purifying SHA homologues from seven strains, the lost strain *Streptomyces* sp. 27S5 turned out to be exceptional, as it expressed and secreted a processed form of SHA protein at 4-fold-higher levels, at least, than the two best SHA homologue-producing strains.

In our previous study, the database search led to the very first hit of an SHA homologue hypothetical protein in the genome of *Streptomyces* sp. Mg1, with 80% amino acid sequence identity to SHA (5). Detection and purification of the SHA homologue from *Streptomyces* sp. Mg1 would not be possible since the homologue does not exhibit HA or carbohydrate binding activities. Anti-SHA reactivity, however, indicated this homologue’s resemblance to SHA.

This study strongly suggests that processing enzymes are secreted from most of the strains which produced SHA homologues and that such processing occurred even after SHA homologues were purified. It is not clear whether such processing enzymes are physically associated with pro-SHA proteins. The processing occurred more rapidly when the purified SHA homologues were kept at 4°C than at −80°C. Potential cleavage sites do not seem to demonstrate clear consensus sequences for associated proteases. Further investigations are obviously required for determining the nature of associated proteases.

Apart from the structural and functional studies of SHA homologue proteins described above, it should be noted that the present study led to an intriguing discovery, that is, the identification of the SHA gene in syntenic regions. The distribution of the SHA gene homologue in *S. lavendulae*-related strains was surveyed among 1,234 *Streptomyces* strains, which were downloaded from NCBI genome databases. The conservation of gene order or synteny among *S. lavendulae*-related strains with or without the SHA gene is evident, as shown in Fig. 8. The syntenic regions consist of around 17 ORFs, which are mostly conserved, with the exception of a few insertions: ORF −11 for some of groups a and d and lipoprotein between ORFs −4 and −5 for group d1 (Table 8). These strongly indicate insertion by horizontal gene transfer (HGT). On 16S rRNA, *gyrB*, and *proB* phylogenetic trees, SHA or LP gene-possessing strains show mosaic distributions, indicating that multiple events of HGT had occurred. Multiple HGT insertions into the same locus, however, appear to be inconsistent. This suggests that the entire or partial HGT in the synteny region is occurring. Syntenic regions must have been preserved by genome rearrangements during evolution. Some strains, in fact, contain an IR (inverted repeat) surrounding their syntenic regions, suggesting HGT events taking place with this syntenic region (8–10). This hypothesis is also supported by a consistent phylogenetic relationship of genes within the synteny region with SHA or LP (Fig. 8C and D). It may also explain the concentrated distribution of the SHA gene in *S. lavendulae*-related strains. That is, the following mechanism can be considered. First, the SHA gene was introduced into an *S. lavendulae*-related strain by an HGT event. This introduction must have happened within this synteny region. Subsequently, multiple HGTs by homologous recombination of this region occurred among *S. lavendulae*-related strains sharing this synteny region. The possibility that HGT by homologous recombination of the synteny region occurs more frequently than by nonhomologous recombination could explain the specific distribution of the SHA gene in *S. lavendulae*-related strains. It could also explain the mosaic distribution of the SHA gene among *S. lavendulae*-related strains. In addition to SHA and LP loci, the loci of ORF −14 and ORF −11 or between ORF −2 and ORF −3 are also considered to be insertion “hot spots.” The syntenic regions must have been preserved by genome rearrangements during evolution. Another interesting observation is that ORF −4 encodes a putative hydrolase. Future experiments will investigate whether this hydrolase is the putative protease that processes pre- or pro-SHA forms.

Although the role of SHA in the syntenic region remains elusive, it is plausible that SHA plays a role in the unknown function achieved by the genes in the synteny. The new finding of the syntenic region in which 8 potential gene products are suggested (Fig. S2) may provide clues to future determination of the role of SHA. It is well known that numerous members of the genus *Streptomyces* produce secondary metabolites such as antibiotics and other pharmacological agents, including neurological agents, immunomodulators, antitumor agents, and enzyme inhibitors. Genome-sequencing projects have revealed that 30 to 36 gene clusters related to the biosynthesis of known or unidentified secondary metabolites exist in *Streptomyces* genomes (11, 12). The identification of biosynthetic gene clusters has provided tools not only for the elucidation of the biosynthesis of secondary metabolites but also for the controlled genetic engineering of these biosynthetic gene clusters for production of secondary metabolites of interest (13, 14). Future studies of the genes in the SHA gene syntenic regions in relation to possible secondary metabolite production may reveal the role of SHA in the life cycle of *S. lavendulae* strains. From another point of view, it is interesting to speculate that these syntenic regions may play an important role in host-pathogen interactions, since lipoproteins apparently have biological properties associated with virulence (15).

The genus *Streptomyces* is most important in ecological function, representing up to 90% of all soil actinomycetes, and therefore the unknown characteristics of SHA may play an important role in the soil actinomycete population. The ecological function of SHA homologues of *Streptomyces* strains is unknown, but in certain circumstances, this gene product may be advantageous for survival. Elucidation of the ecological and biological significance of SHA homologue products will remain an important future topic. In conclusion, this study has provided the basis for understanding how SHA is secreted and processed, monovalent to divalent-HA active form, and how SHA plays a role in nature.

## MATERIALS AND METHODS

### Bacterial strains.

From the IMC collection of 40,000 actinomycete strains, partial 16S rRNA sequences of 5,000 *Streptomyces* strains with single-pass DNA sequencing data by PCR were readily available. Of those, 67 strains with 99.6% to 100% 16S rRNA sequence homology to that of *S. lavendulae* were selected (see Table S2 in the supplemental material). Ten strains were selected from WB-positive or pseudopositive strains to cover a wide variety of 16S rRNA gene phylogeny. The two *S. lavendulae* strains used in this study were strain ATCC 14158, abbreviated *Lav*, purchased by City of Hope, and the ISP5069 strain kept at IMC in Japan (abbreviated strain #27), originating from S. A. Waksman IMRU 3440-8 and essentially the same as ATCC 14158 but appearing to be a separate clone.

### Culture conditions.

All chemicals used were from Fujifilm Wako Pure Chemical Corporation, Osaka, Japan, unless otherwise stated. A medium containing d-galactose (20 g), dextrin (20 g), soy peptone (Life Technologies Corporation, Detroit, MI) (10 g), corn steep liquor (Kogostch Co., Ltd., Chiba, Japan) (5 g), and (NH_4_)_2_SO_4_ (2 g) in 1 liter of tap water (pH 7) was autoclaved and used for seed cultures. A 500-ml baffled Erlenmeyer flask containing 110 ml of the seed culture medium was supplemented with CaCO_3_ (220 mg) and 1 drop of silicon antiforming agent, a mixture of KM-70 (Shin-Etsu Chemical Co. Ltd. Tokyo, Japan) and soybean oil (1:1). After inoculation of *Streptomyces* strains, flasks were incubated at 27°C for 3 days at 180 rpm. Two percent of each seed culture was added to 110 ml of the main culture in each 500-ml baffled Erlenmeyer flask. The main culture medium consisted of sterilized High Polypeptone (5 g), Bacto Casamino Acids (Life Technologies Corporation) (5 g), d-mannose (10 g), and MgSO_4_·7H_2_O (0.5 g) in 1 liter of tap water. The main culture was continued by incubation at 27°C for 4 to 5 days at 180 rpm.

### 16S rRNA gene sequencing.

Single-pass sequences (∼500 bases in length) of IMC strains were determined following colony PCR as previously described (16) using primer 9f listed in Table 1. For phylogenetic tree analysis of 16S rRNA genes, almost full-length 16S rRNA genes (∼1,500 bases/strain) were amplified by colony PCR with primers 16S001F and 16S003R. Respective 16S rRNA gene sequences were determined by combination of sequencing data using 9f, 338F, 536/517R, and 907/928F. Phylogenetic trees were constructed with ClustalW software to clarify phylogenetic relationships (17).

### PCR screening of SHA gene homologues and determination of DNA sequences of 10 SHA homologues.

Colony PCR of 67 strains was performed with two primer sets, sets g and i, consisting of SHA61f/SHA4r and SHA13f/SHA32r, respectively (Fig. 1; Table 1). The presence or absence of amplification was confirmed by agarose gel electrophoresis.

PCR products of primer set g were used for the determination of SHA gene homologue sequences of all strains listed in Table 2, except for strain #26. To determine the DNA sequence of SHA homologues, SHA13f, SHA32r, SHA5f, and SHA24r, listed in Table 1, were used as sequencing primers. Because strain #26 gave only a faint PCR product with primer set g, SHA63f and SHA24r26 were used instead of SHA61f and SHA24r, respectively.

### Preparation of anti-SHA.

Rabbit polyclonal antibodies against SHA were raised using the authentic archived SHA with the aid of the Support Unit for Biomaterial Analysis and Animal Resources Development at the RIKEN BSI Research Resources Center. Two rabbits (Japanese white Kbl:JW) were inoculated subcutaneously on the back with 1 mg of SHA each. After 2 and 4 weeks, rabbits received second and third immunizations, respectively, each with the same amounts of SHA. Freund's complete adjuvant H37Ra (catalog no. 231131) was used for first and second immunizations, while Freund's incomplete adjuvant (catalog no. 263910) (Difco Laboratories) was used for the third immunization. At 1 week after the third immunization, sera were collected from the rabbits. ELISA demonstrated that sera from both rabbits showed good positivity against SHA even at 25,600-fold dilutions.

### Western blotting of culture supernatants.

Culture supernatants of 67 strains were collected by centrifugation at 12,000 × *g* for 20 min at 4°C. Culture supernatants (200 μl) were concentrated 4-fold to 50 μl following TCA/acetone protein precipitation prior to WB. Briefly, 200 μl of 60% TCA was mixed with an equal volume of culture supernatant and left for 30 min on ice. The mixture was then centrifuged at 17,200 × *g* for 15 min at 4°C. Supernatants were carefully removed, and the pellets were washed twice with 200 μl of cold acetone and centrifuged for 10 min at 4°C. The pellets were air-dried and dissolved in 50 μl of SDS-PAGE sample buffer containing 0.5M Tris(2-carboxyethyl)phosphine (TCEP). Samples were heated at 95°C for 5 min, and 15 μl of each was loaded into a well of precast gels (NuPAGE 4-to-12% bis-Tris protein gels, 1.0 mm, 15 wells; Invitrogen). Precision Plus Protein Kaleidoscope standards (Bio-Rad catalog no. 161-0375) and the authentic SHA were used as a molecular standard and a positive control, respectively. After electrophoresis was completed, proteins were transferred to a polyvinylidene difluoride (PVDF) membrane (Immobilon-FL PVDF membrane; Millipore) at 23 V and 300 W for 1 h 40 min. The membranes were blocked with blocking buffer (TBS Odyssey blocking buffer; LI-COR) at 4°C overnight. The membranes were incubated with rabbit anti-SHA (1:250 dilution) for 2 to 3 h at room temperature and then washed with phosphate-buffered saline (PBS) containing 0.1% Triton-X for 5 min, 4 times. Donkey anti-rabbit IgG secondary antibody (IRDye 680LT; LI-COR Biosciences) was added at a 1:5,000 dilution and incubated for 1 h at room temperature. The images were visualized using a LI-COR Odyssey 9120 imaging system.

### Purification of SHA homologues from six *Streptomyces* strains.

Cells were grown in 110 ml each in a 500-ml baffled Erlenmeyer flask for seed and main cultures as described above. Culture broths from 18 flasks were combined and centrifuged at 12,000 × *g* for 20 min at 4°C to prepare 2-liter culture supernatants from 6 strains. Purification of SHA homologues was carried out as published previously (3). Briefly, to each 2-liter culture supernatant, 7 ml of gum arabic gels (50% suspension) was added and incubated at 4°C overnight with stirring. Gels were collected by centrifugation and washed with PBS 2 to 3 times. SHA analogues were eluted from the gels in 50-ml tubes by the addition of 1.5 ml of 0.2 M l-rhamnose containing 1 M NaCl, followed by mixing on a rotator for 30 min at 4°C and centrifugation at 3,900 × *g* for 10 min. After the first collection of supernatants in fraction 1, fractions 2 to 5 were obtained in the same manner, except that the gels were eluted with 1 ml of 0.2 M l-rhamnose containing 1 M NaCl per fraction. SDS-PAGE analysis of five stepwise-eluted fractions corresponding to six strains is shown in Fig. 3A. Originally, stepwise elution was carried out to avoid packing of gels, which could interfere with the elution of SHA homologues. Purification was further carried out by column elution methods to recover the SHA homologues that still remained in the gum arabic gels. Fractions containing SHA homologues were concentrated using Amicon Ultra-15 centrifugal filter units with a 10-kDa cutoff (Millipore Sigma) at 4°C, 3,900 × *g*, for 15 min in volumes ranging from 0.3 to 1 ml. Protein concentrations of the concentrated SHA homologues were estimated by either the bicinchoninic acid (BCA) or Bradford assay (Thermo Fisher Scientific). When needed, NanoDrop or densitometric scanning was carried out to determine protein amounts. Two to five micrograms of protein per lane was analyzed by SDS-PAGE as described above. Protein bands were visualized using SimplyBlue safe stain (Thermo Fisher Scientific).

### Hemagglutination assays.

Hemagglutination assays were performed as previously described (3). Human blood samples were collected from the City of Hope Blood Donor Center under approved IRB protocol no. 99132. Briefly, hemagglutinating activity was measured on a 96-well microtiter plate (U bottom) using a 2% human A, B, or O erythrocyte suspension and PBS as a diluent. Hemagglutination reaction was carried out for 90 min at room temperature. Activity was expressed as a titer, the reciprocal of the highest 2-fold dilution exhibiting positive hemagglutination. Due to the nature of this 2-fold dilution assay, a 2-fold difference is considered within possible error.

### Determination of molecular masses of purified SHA analogues by Q-TOF mass spectrometry.

Molecular masses of purified SHA analogues were determined using an Agilent 6520 accurate-mass quadrupole time-of-flight (Q-TOF) LC/MS system (Agilent, Santa Clara, CA, USA) with Agilent MassHunter qualitative analysis software. Liquid chromatography of each SHA homologue sample (2 to 5 μl) was performed using an Agilent HPLC-Chip G4240-63001. The HPLC buffer A/B gradient system used was as follows: 0 to 8 min, from 1.0% to 90.0% B; 8.0 to 9.0 min, from 90.0% to 90.0% B; 9 to 11 min, from 90% to 1% B. Buffer A was 0.1% formic acid, and buffer B was 0.1% formic acid in acetonitrile. The BSA standard and a blank were measured before each sample was injected to ensure data quality.

### Mass spectrometric analyses of the 7 purified SHA homologues.

Concentrated samples of SHA homologues in PBS, pH 7.4, were separated on NuPAGE 4-to-12% bis-Tris protein gels (Thermo Fisher Scientific) as described above (Fig. 3A), followed by an in-gel digestion for mass spectrometric analyses. To extract peptides from the SHA homologues (approximately 1 to 3 μg), gel bands were digested separately with trypsin/LysC (Promega) or chymotrypsin (Promega), generating various overlapping peptides as described below. The peptides were analyzed by LC coupled with high-resolution multistage mass spectrometry (MS and MS/MS).

In more detail, the singular bands were cut and destained in 50 to 100 μl of 45% acetonitrile–55% 100 mM ammonium bicarbonate (vol/vol) and incubated at 4°C overnight. The proteins were then reduced and alkylated by shrinking in 100% acetonitrile and treatment with 50 to 100 μl of 10 mM TCEP in 100 mM ammonium bicarbonate at 37°C for 30 min. The supernatant was discarded, and 20 μl of 50 mM iodoacetamide in 100 mM ammonium bicarbonate was added to the sample. It was then incubated in the dark for 1 h at room temperature. The samples were washed by adding 50 to 100 μl of acetonitrile and then with 50 μl of 50 mM ammonium bicarbonate. The cut gel pieces were then shrunk again in 50 μl of acetonitrile.

Each enzyme digestion was carried out basically in accordance with the manufacturer's instructions. Approximately 60 ng of trypsin-LysC mix (Trypsin Gold, mass spectrometry grade, and rLys-C, mass spectrometry grade; Promega) was added to the protein samples in 200 μl of 50 mM ammonium bicarbonate and incubated at 37°C overnight. Chymotrypsin (bovine pancreas; Worthington Biochemical Corporation, Lakewood, NJ) was reconstituted in 1 mM HCl, 25 to 50 μl of which was used per digestion to make a final concentration of 0.5 to 1 μg/μl. Approximately 50 ng of the enzyme was added to the samples in 200 μl of 50 mM ammonium bicarbonate and incubated at 37°C overnight. In addition, Arg-C (Promega) and pepsin (Sigma-Aldrich) digestions were performed for strain #26 and *Lav* SHA homologues. Arg-C was suspended in 50 mM Tris-HCl, pH 7.8, containing 5 mM CaCl_2_ and 2 mM EDTA. Once approximately 50 ng of the enzyme had been added to the gel pieces, dithiothreitol (DTT) was added to the sample at a final concentration of 2 mM to activate the enzyme in a total volume of 200 μl. Samples were incubated at 37°C overnight. Prior to pepsin digestion, the destained bands were mixed with Milli-Q water at pH 2 (acidified with 10 mM HCl). Pepsin was suspended at 0.1 μg/μl in double-distilled water at pH 6.0. About 50 ng of pepsin was added to each sample and incubated at 37°C overnight. To stop the reaction, the samples were heated at 95°C.

To extract the digested peptides from the gel pieces, 1% formic acid (100 μl) was added to the gel pieces and incubated at 37°C for 30 min. After the supernatants were collected, 50% acetonitrile–1% formic acid was added to the gel pieces and incubated for another 30 min. Combined supernatants were lyophilized and reconstituted in 25 to 50 μl of 1% formic acid. To ensure that the enzymatic digests are free of contaminants, SPE (solid-phase extraction) purification was conducted using Oasis HLB 1-cc Sep-Pak columns (Waters Corporation, MA, USA). Each column was conditioned with 2 volumes of 100% ethanol, followed by Milli-Q water, and finally with 0.1% trifluoroacetic acid (TFA) in water with a vacuum suction device. Once a column was equilibrated, the sample was loaded, and it was washed with 2 volumes of 0.1% TFA in water; then, the column was allowed to dry for 1 min under vacuum. Peptides were then eluted with 2 volumes of 70% acetonitrile–0.1% TFA. The eluents were lyophilized. Once dried, the peptides were reconstituted in 200 μl of 0.1% formic acid and transferred to mass spectrometry tubes for LC-MS/MS analysis. Peptides were analyzed by LC-MS on an Orbitrap Fusion Tribrid mass spectrometer (Thermo Fisher Scientific). MS and MS/MS collision-induced dissociation fragmentation data from these peptides were analyzed with Xcalibur software (Thermo Fisher Scientific) and with PEAKS Studio software (Bioinformatics Solutions Inc., Waterloo, Canada).

### BLAST search, ORF prediction, and phylogenetic tree construction.

Genome sequence data were downloaded from the NCBI genome site (https://www.ncbi.nlm.nih.gov/genome). A local BLAST system was constructed with command-line tools of BLAST+ downloaded from the NCBI BLAST site (https://blast.ncbi.nlm.nih.gov/Blast.cgi) (18). ORFs were predicted by FramePlot 4.0beta (http://nocardia.nih.go.jp/fp4/) (19). Phylogenetic trees were constructed by the neighbor-joining method (20) using the ClustalW package (21) on the DDBJ site (https://www.ddbj.nig.ac.jp/index.html).
