# Supplementary material for: Proteolytic Processing, Maturation, and Unique Synteny of the Streptomyces Hemagglutinin SHA
Source: Microbiol Spectr. 2021 Sep 1;9(2):e00766-21. doi: 10.1128/Spectrum.00766-21 (PMC8557816; doi:10.1128/Spectrum.00766-21)
Supplement: SUPPLEMENTAL FILE 1 — Supplemental material. Download SPECTRUM00766-21_Supp_1_seq1.pdf, PDF file, 0.9 MB. [file spectrum00766-21_supp_1_seq1.pdf]

Supplemental Material for:

"Proteolytic Processing, Maturation, and Unique Synteny of the *Streptomyces* Hemagglutinin SHA"

Running title: Biochemistry and Genetics of the Microbial Lectin SHA

Authors and affiliations:

Yoko Fujita-Yamaguchi[a, b, #], Hideyuki Muramatsu[c], Alonso Tapia[b], Karine Bagramyan[b], Moksha Desai[b], Yasuhiro Takehana[c], Masayuki Igarashi[c], Yoshiki Yamaguchi[d], and Markus Kalkum[b, #]

[a] Department of Diabetes Complications & Metabolism, Arthur Riggs Diabetes and Metabolism Research Institute, Beckman Research Institute, City of Hope, Duarte, CA

[b] Department of Immunology & Theranostics, Arthur Riggs Diabetes and Metabolism Research Institute, Beckman Research Institute, City of Hope, Duarte, CA

[c] Laboratory of Microbiology, Institute of Microbial Chemistry, Tokyo, Japan

[d] Biomolecular Characterization Unit, RIKEN Center for Sustainable Resource Science, RIKEN, Saitama, Japan

[#] Address correspondence to: Yoko Fujita-Yamaguchi, yyamaguchi@coh.org, and Markus Kalkum, mkalkum@coh.org

[d] Current affiliation of Yoshiki Yamaguchi: Laboratory of Pharmaceutical Physical Chemistry, Tohoku Medical and Pharmaceutical University

Figure S1

A. #9

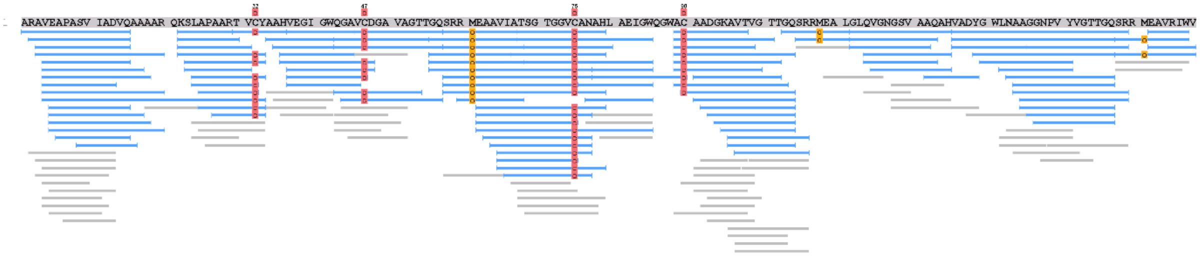

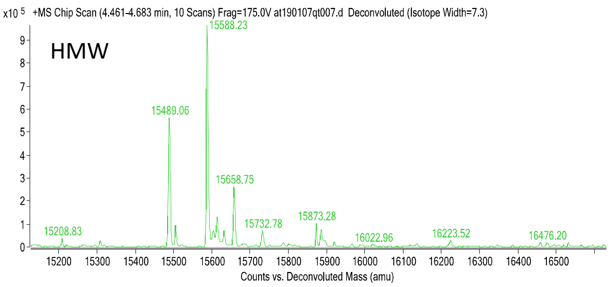

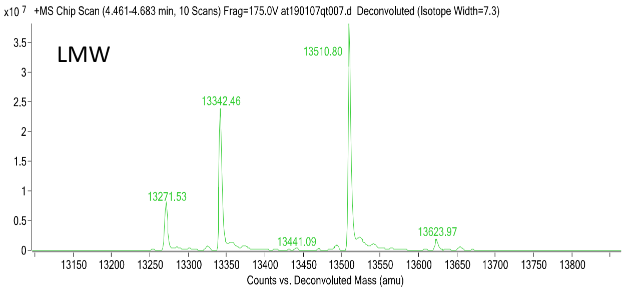

15,658.40 Da
15,587.32 Da
15,488.19 Da

13,509.98 Da
13,342.46 Da
13,270.71 Da

calculated masses and AA sequence

1 ARAVEAPASVIADVQAAAARQKSLAPAARTVCYAAHVEGIGWQGAVCDGAVAGTTGQSRRMEAAVIATSGTGGVCANAHLAE

81 IGWQGWACAADGKAVTVGTTGQSRRMEALGLQVGNGSVAAQAHVADYGWLNAAGGNPVYVGTTGQSRRMEAVRIWV

B. #19

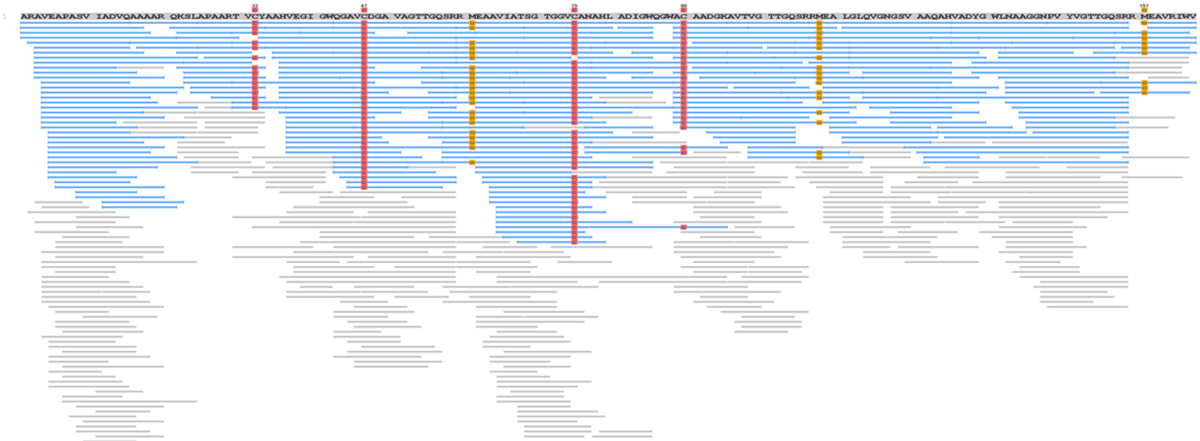

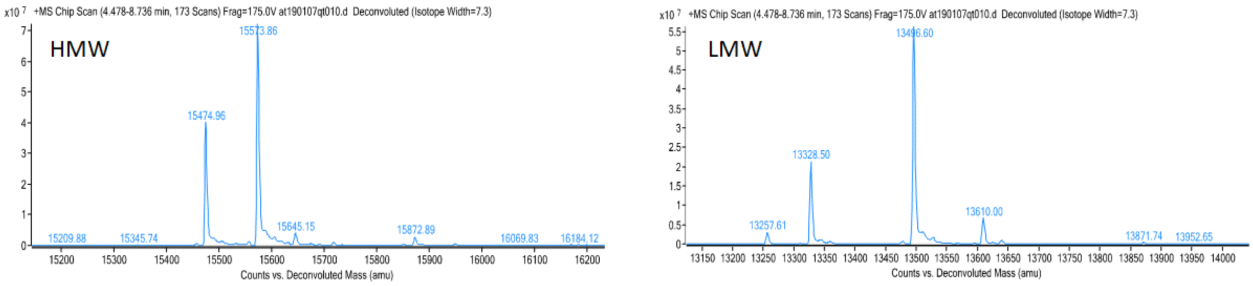

→15,871.64 Da
→15,573.29 Da
→15,474.16 Da
→13,609.11 Da
→13,495.95 Da
→13,327.76 Da

calculated masses
and AA sequence

1 ARAVEAPASVIADVQAAAARQKSLAPAARTVCYAAHVEGIGWQGAVCDGAVAGTTGQSRRMEAAVIATSGTGGVCANAHL

81 ADIGWQGWACAADGKAVTVGTTGQSRRMEALGLQVGNGSVAAQAHVADYGWLNAAGGNPVYVGTTGQSRRMEAVRIWV

C. #38

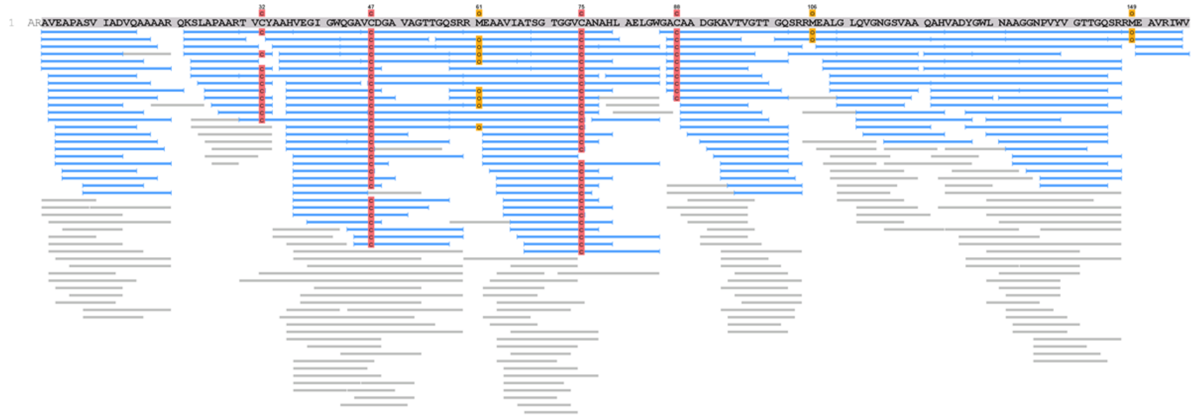

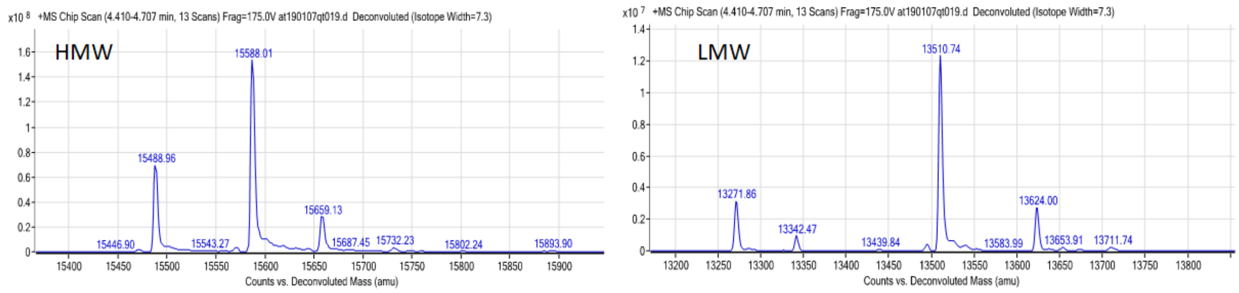

→15,658.40 Da
→15,587.32 Da
→15,488.19 Da
→13,623.14 Da
→13,509.98 Da
→13,342.46 Da
→13,270.71 Da

calculated masses
and AA sequence

1 ARAVEAPASVIADVQAAAARQKSLAPAARTVCYAAHVEGIGWQGAVCDGAVAGTTGQSRRMEAAVIATSGTGGVCANAHLAE

81 IGWQGWACAADGKAVTVGTTGQSRRMEALGLQVGNGSVAAQAHVADYGWLNAAGGNPVYVGTTGQSRRMEAVRIWV

D. #57

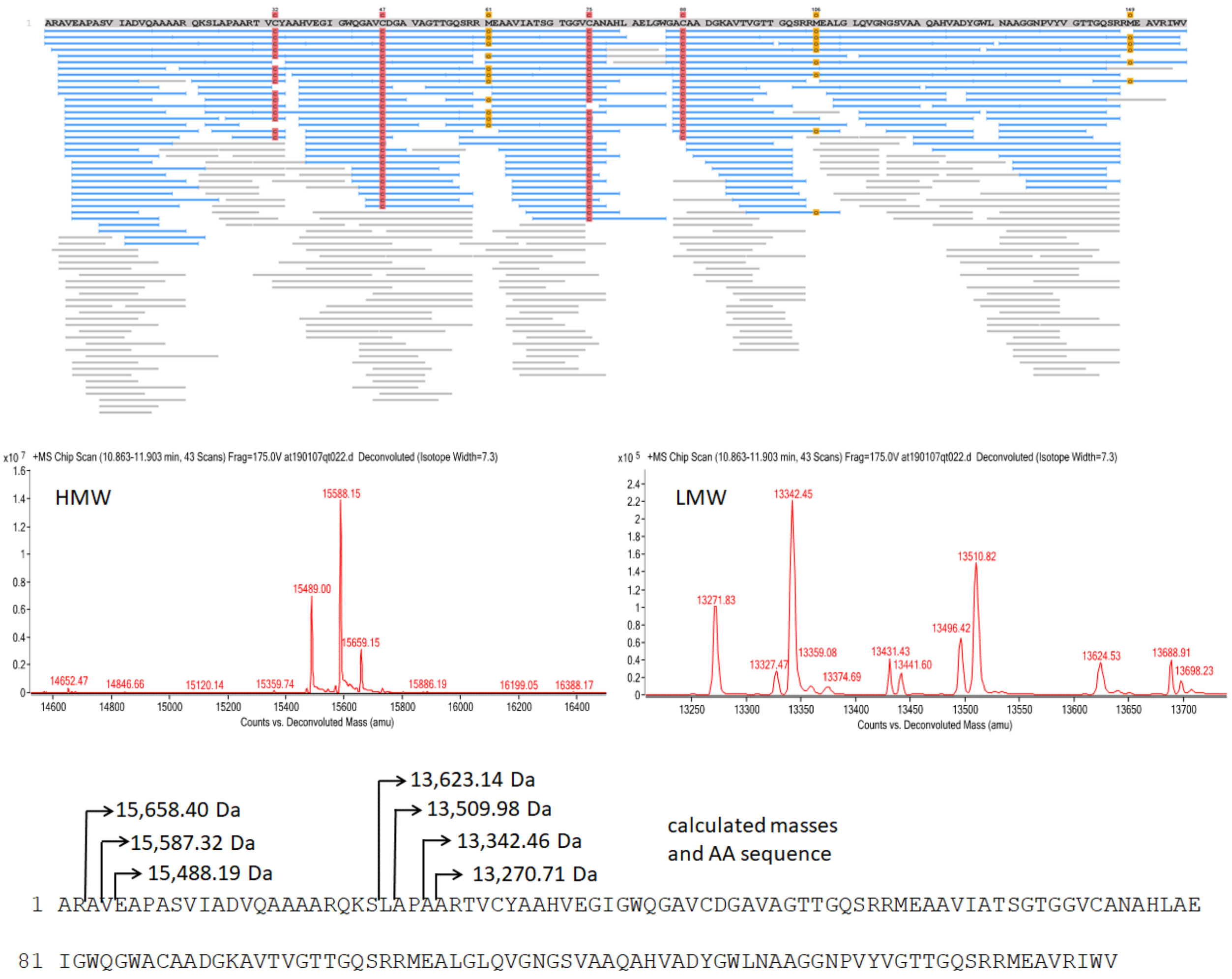

Figure S1 legend

<u>Maturation of SHA in strains #9 (A), #19 (B), #38 (C), and #57 (D).</u>

<u>Top panels</u>: LC-MS/MS analyses of tryptic and chymotryptic peptides, derived from respective bands seen in Figure 3A, aligned to the deduced amino acid sequence determined by DNA sequencing of the strain-specific SHA gene. Blue bars indicate database-matched peptides, grey bars mark *de novo* sequencing results. Carbamidomethylated cysteine residues are highlighted in red.

<u>Middle panels</u>: Deconvoluted ESI Q-TOF MS spectra of high and low molecular weight mass regions (HMW and LMW) corresponding to pro-SHA and SHA, respectively.

<u>Bottom panels</u>: Amino acid sequences for SHA precursors of strains #9, #19, #38, and #57 with start sites of pro-SHA and mature SHA proteins marked based on the molecular masses determined by Q-TOF MS.

Figure S2.

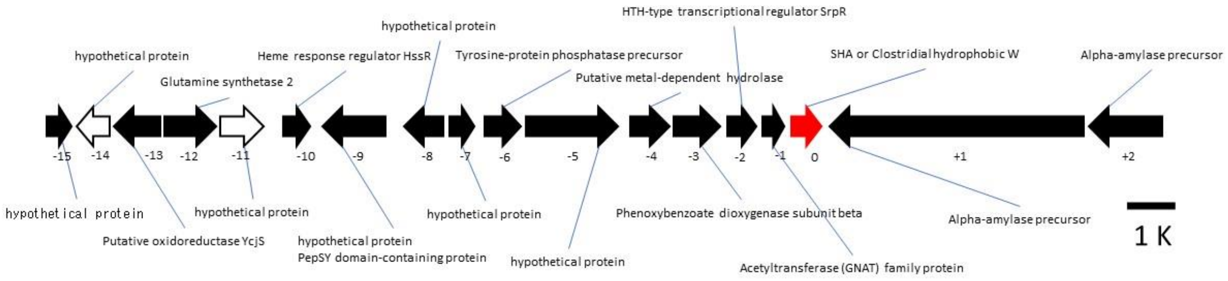

	CP024985.1		
	locus_tag	locus	product
orf(-15)	SLAV_26185	complement(5876178..5876750)	hypothetical protein
orf(-14)	SLAV_26180	5875391..5876164	hypothetical protein
orf(-13)	SLAV_26175	5874391..5875383	putative oxidoreductase YcjS
orf(-12)	SLAV_26170	complement(5873238..5874266)	Glutamine synthetase 2
orf(-11)	SLAV_26165	complement(5872194..5873162)	hypothetical protein
orf(-10)	SLAV_26160	complement(5871234..5871836)	Heme response regulator HssR
orf(-9)	SLAV_26155	5869461..5871116	hypothetical protein
orf(-8)	SLAV_26150	5868513..5869382	hypothetical protein
orf(-7)	SLAV_26145	complement(5867830..5868330)	hypothetical protein
orf(-6)	SLAV_26140	complement(5866897..5867691)	Tyrosine-protein phosphatase precursor
orf(-5)	SLAV_26135	complement(5864888..5866810)	hypothetical protein
orf(-4)	SLAV_26130	complement(5863805..5864701)	putative metal-dependent hydrolase
orf(-3)	SLAV_26125	complement(5862753..5863808)	Phenoxybenzoate dioxygenase subunit beta
orf(-2)	SLAV_26120	complement(5861996..5862703)	HTH-type transcriptional regulator SrpR
orf(-1)	SLAV_26115	complement(5861441..5861965)	Acetyltransferase (GNAT) family protein
orf(-0)	SLAV_26110	complement(5860685..5861296)	Clostridial hydrophobic W
orf(+1)	SLAV_26105	5855278..5860620	Alpha-amylase precursor
orf(+2)	SLAV_26100	5853515..5855233	Alpha-amylase precursor

Figure S2 legend

The syntenic region consisting of orf (-15) to orf (+2) as seen in *Streptomyces lavendulae* subsp. *lavendulae* CCM 3239.

The current study led to an intriguing discovery, that is, the identification of the SHA gene in syntenic regions. Distribution of the SHA homologue gene in *S. lavendulae*-related strains was surveyed among 1,234 Streptomyces strains, which were obtained from NCBI Genome databases. The conservation of gene order or synteny among *S. lavendulae*-related strains with or without the SHA gene is evident as shown in Fig. 8. The syntenic regions consist of around 17 orfs, which are mostly conserved with the exception of a few insertions; orf (-11) for some of group a, and d and lipoprotein between orfs (-4) and (-5) for group d1 (Table 8).

Table S1. List of 67 *Streptomyces* strains from the IMC collection

	Strain ID	Closest species (16S rRNA gene homology %)	Source
1	3007-11	*Streptomyces vinaceus* NBRC 13425 (99.87)	Soil (Tokyo, JAPAN)
2	3007-35	*Streptomyces spororaveus* LMG 20313 (100)	Soil (Tokyo, JAPAN)
3	3009-15	*Streptomyces virginiae* NBRC 12827 (100)	Soil (Tokyo, JAPAN)
4	3009-45	*Streptomyces vinaceus* NBRC 13425 (100)	Soil (Shizuoka, JAPAN)
5	3009-66	*Streptomyces vinaceus* NBRC 13425 (100)	Soil (Niigata, JAPAN)
6	3010-61	*Streptomyces spororaveus* LMG 20313 (100)	Soil (Tokyo, JAPAN)
7	3011-71	*Streptomyces sporoverrucosus* NRRL B-16379T (100)	Soil (Tokyo, JAPAN)
8	3011-72	*Streptomyces sporoverrucosus* NRRL B-16379T (100)	Soil (Tokyo, JAPAN)
9	3015-22	*Streptomyces sporoverrucosus* NRRL B-16379T (100)	Soil (Aomori, JAPAN)
10	3015-50	*Streptomyces vinaceus* NBRC 13425 (99.75)	Soil (Nara, JAPAN)
11	3015-60	*Streptomyces tanashiensis* IFO 12919 (100)	Soil (Nagano, JAPAN)
12	3015-62	*Streptomyces vinaceus* NBRC 13425 (99.87)	Soil (Nagano, JAPAN)
13	3016-25	*Streptomyces spororaveus* LMG 20313 (100)	Soil (Akita, JAPAN)
14	3016-29	*Streptomyces spororaveus* LMG 20313 (100)	Soil (Akita, JAPAN)
15	3017-08	*Streptomyces spororaveus* LMG 20313 (100)	Soil (Tokyo, JAPAN)
16	3017-16	*Streptomyces spororaveus* LMG 20313 (100)	Soil (Tokyo, JAPAN)
17	3020-26	*Streptomyces sporoverrucosus* NRRL B-16379T (99.87)	Soil (JAPAN)
18	3020-41	*Streptomyces vinaceus* NBRC 13425 (100)	Soil (JAPAN)
19	3024-17	*Streptomyces spororaveus* LMG 20313 (100)	Soil (Nagano, JAPAN)
20	3024-67	*Streptomyces spororaveus* LMG 20313 (99.87)	Soil (Nagano, JAPAN)
21	3025-21	*Streptomyces sporoverrucosus* NRRL B-16379T (100)	Soil (Nagano, JAPAN)
22	5005-65	*Streptomyces cavourensis* subsp. *cavourensis* NBRC 13026 (99.88)	Leaf litter (OKinawa, JAPAN)
23	isolate 135	*Streptomyces vinaceus* NBRC 13425 (100)	Leaf litter (Ibaraki, JAPAN)
24	isolate 474	*Streptomyces virginiae* NBRC 12827 (100)	Leaf litter (Tokyo, JAPAN)
25	isolate 484	*Streptomyces vinaceus* NBRC 13425 (99.74)	Leaf litter (Tokyo, JAPAN)
26	isolate 491	*Streptomyces vinaceus* NBRC 13425 (99.74)	Leaf litter (Tokyo, JAPAN)
27	ISP 5069 (= ATCC 14158)	*Streptomyces lavendulae* subsp. *lavendulae*	(type strain)
28	ISP 5190	*Streptomyces goshikiensis*	(type strain)
29	ISP 5094	*Streptomyces virginiae*	(type strain)
30	ISP 5134	*Streptomyces xanthophaeus*	(type strain)
31	BSA00069	*Streptomyces spororaveus* LMG 20313 (100)	Soil (JAPAN)
32	BSA00096	*Streptomyces spororaveus* LMG 20313 (99.87)	Soil (JAPAN)
33	BSA00115	*Streptomyces spororaveus* LMG 20313 (100)	Soil (JAPAN)
34	BSA00390	*Streptomyces spororaveus* LMG 20313 (100)	Soil (JAPAN)

35	BSA00522	*Streptomyces cavourensis* subsp. *cavourensis* NBRC 13026 (99.88)	Soil (JAPAN)
36	BSA00604	*Streptomyces vinaceus* NBRC 13425 (99.86)	Soil (JAPAN)
37	BSA00612	*Streptomyces spororaveus* LMG 20313 (100)	Soil (JAPAN)
38	BSA00614	*Streptomyces spororaveus* LMG 20313 (100)	Soil (JAPAN)
39	BSA00653	*Streptomyces spororaveus* LMG 20313 (100)	Soil (JAPAN)
40	BSA00721	*Streptomyces sporoverrucosus* NRRL B-16379 (99.87)	Soil (JAPAN)
41	BSA00724	*Streptomyces vinaceus* NBRC 13425 (99.62)	Soil (JAPAN)
42	BSA00750	*Streptomyces spororaveus* LMG 20313 (100)	Soil (JAPAN)
43	BSA00754	*Streptomyces cavourensis* subsp. *cavourensis* NBRC 13026 (100)	Soil (JAPAN)
44	BSA00788	*Streptomyces vinaceus* NBRC 13425 (100)	Soil (JAPAN)
45	BSA00879	*Streptomyces spororaveus* LMG 20313 (100)	Soil (JAPAN)
46	BSA00897	*Streptomyces sporoverrucosus* NRRL B-16379 (100)	Soil (JAPAN)
47	BSA00904	*Streptomyces spororaveus* LMG 20313 (99.86)	Soil (JAPAN)
48	BSA00928	*Streptomyces sporoverrucosus* NRRL B-16379 (99.86)	Soil (JAPAN)
49	BSA00970	*Streptomyces spororaveus* LMG 20313 (100)	Soil (JAPAN)
50	BSA00995	*Streptomyces spororaveus* LMG 20313 (100)	Soil (JAPAN)
51	BSA01022	*Streptomyces sporoverrucosus* NRRL B-16379 (100)	Soil (JAPAN)
52	BSA01058	*Streptomyces lavendulae* subsp. *lavendulae* NBRC 12789 (99.73)	Soil (JAPAN)
53	BSA01081	*Streptomyces sporoverrucosus* NRRL B-16379 (100)	Soil (JAPAN)
54	BSA01199	*Streptomyces sporoverrucosus* NRRL B-16379 (100)	Soil (JAPAN)
55	BSA01223	*Streptomyces colombiensis* NRRL B-1990 (100)	Soil (JAPAN)
56	BSA01230	*Streptomyces sporoverrucosus* NRRL B-16379 (100)	Soil (JAPAN)
57	BSA01231	*Streptomyces spororaveus* LMG 20313 (100)	Soil (JAPAN)
58	BSA01241	*Streptomyces sporoverrucosus* NRRL B-16379 (99.73)	Soil (JAPAN)
59	BSA01311	*Streptomyces cavourensis* subsp. *cavourensis* NBRC 13026 (99.73)	Soil (JAPAN)
60	BSA01346	*Streptomyces spororaveus* LMG 20313 (100)	Soil (JAPAN)
61	BSA01347	*Streptomyces sporoverrucosus* NRRL B-16379 (99.87)	Soil (JAPAN)
62	BSA01351	*Streptomyces vinaceus* NBRC 13425 (99.73)	Soil (JAPAN)
63	BSA01474	*Streptomyces spororaveus* LMG 20313 (100)	Soil (JAPAN)
64	BSA03079	*Streptomyces vinaceus* NBRC 13425 (99.87)	Soil (JAPAN)
65	BSA03107	*Streptomyces spororaveus* LMG 20313 (100)	Soil (JAPAN)
66	BSA03224	*Streptomyces vinaceus* NBRC 13425 (99.87)	Soil (JAPAN)
67	BSA03598	*Streptomyces spororaveus* LMG 20313 (99.86)	Soil (JAPAN)

Table S2. List of full names, accession numbers, and % homology to *S. lavendulae* 16S RNA of the strains shown in Table 8.

Strain	Accession	homology(%) to CCM 3239
S. amritsarensis MTCC 11845^T	NZ_MQUR00000000	98.9%
S. katrae NRRL B-16271	NZ_JNZY00000000	99.8%
S. katrae NRRL ISP-5550^T	NZ_JZWV00000000	98.5%
S. katrae S3	NZ_CP020042	98.6%
S. lavendulae subsp. *lavendulae* CCM 3239	NZ_CP024985	-
S. lavendulae subsp. *lavendulae* NRRL B-2774	NZ_JOEW00000000	100%
S. subrutilus ATCC 27467^T	NZ_CP023701	99.7%
S. venezuelae ATCC 21018	NZ_CP029189	99.9%
S. venezuelae ATCC 21782	NZ_CP029190	99.2%
S. vinaceus ATCC 27476^T	NZ_CP023692	99.8%
S. xanthophaeus NRRL B-5414^T	NZ_JOFT00000000	99.8%
Streptomyces sp. 3211	NZ_CP020039	99.9%
Streptomyces sp. 3211.6	NZ_RBXC00000000	98.6%
Streptomyces sp. A0592	NZ_SSBM00000000	99.5%
Streptomyces sp. A1136	NZ_SSBJ00000000	99.7%
Streptomyces sp. A1547	NZ_SSBH00000000	99.8%
Streptomyces sp. ADI91-18	NZ_RPGS00000000	N.D.
Streptomyces sp. ADI95-16	CP033581	99.7%
Streptomyces sp. Ag109_G2-1	NZ_PHUB00000000	98.6%
Streptomyces sp. Ag109_G2-6	NZ_RKRC00000000	98.6%
Streptomyces sp. BK038	NZ_SLZC00000000	99.8%
Streptomyces sp. CB00455	NZ_LIVQ00000000	99.6%
Streptomyces sp. CB02120-2	NZ_NNBM00000000	99.8%
Streptomyces sp. CB03578	NZ_LWLD00000000	99.8%
Streptomyces sp. CG 926	NZ_QGGZ00000000	99.8%
Streptomyces sp. fd1-xmd	NZ_CP019798	98.9%
Streptomyces sp. IGB124	NZ_LGDH00000000	99.6%
Streptomyces sp. IMTB 1903	NZ_LQYB00000000	98.9%
Streptomyces sp. KS 21	NZ_SOCC00000000	99.8%
Streptomyces sp. Mg1	NZ_ABJF00000000	99.9%
Streptomyces sp. MJM1172	NZ_LIVP00000000	99.9%

Streptomyces sp. NRRL F-2580	NZ_JOIR00000000	99.8%
Streptomyces sp. NRRL F-2664	NZ_JOFX00000000	98.9%
Streptomyces sp. NRRL F-2747	NZ_JOIS00000000	99.8%
Streptomyces sp. NRRL F-4428	NZ_JYJI00000000	98.9%
Streptomyces sp. NRRL F-4474	NZ_JOIB00000000	98.1%
Streptomyces sp. PCS3-D2	NZ_JDUZ00000000	98.8%
Streptomyces sp. SDr-06	NZ_QOLA00000000	98.1%
Streptomyces sp. Sge12	NZ_CP020555	99.9%
Streptomyces sp. TN58	NZ_CP018870	98.9%
Streptomyces sp. W1SF4	NZ_CP034350	98.9%
Streptomyces sp. WAC07061	NZ_RPRS00000000	98.9%
Streptomyces sp. WAC07149	NZ_RQJC00000000	99.7%
Streptomyces sp. WAC5950	NZ_VHQM00000000	99.6%
Streptomyces sp. WM4235	NZ_LGDE00000000	99.6%
Streptomyces sp. WM6368	NZ_LGDA00000000	99.9%
Streptomyces sp. WM6378	NZ_LGDD00000000	98.1%
